# Integrated Framework of the Immune-Defense Transcriptional Signatures in the Arabidopsis Shoot Apical Meristem

**DOI:** 10.3390/ijms21165745

**Published:** 2020-08-11

**Authors:** Muhammad Naseem, Özge Osmanoğlu, Martin Kaltdorf, Afnan Ali M. A. Alblooshi, Jibran Iqbal, Fares M. Howari, Mugdha Srivastava, Thomas Dandekar

**Affiliations:** 1 Department of Life and Environmental Sciences, College of Natural and Health Sciences, Zayed University, PO Box 144534-D, Abu Dhabi 4783, UAE; afnan.alblooshi@outlook.com (A.A.M.A.A.); Jibran.Iqbal@zu.ac.ae (J.I.); Fares.Howari@zu.ac.ae (F.M.H.); 2 Department of Bioinformatics, Biocenter, University of Würzburg, Am Hubland, D-97074 Würzburg, Germany; oezge.osmanoglu@uni-wuerzburg.de (Ö.O.); m.kaltdorf@gmx.de (M.K.); mugdha.srivastava@uni-wuerzburg.de (M.S.)

**Keywords:** defense signaling, shoot apical meristem, CLV3p, meta-transcriptome, system inference, stem-cell-triggered immunity

## Abstract

The growing tips of plants grow sterile; therefore, disease-free plants can be generated from them. How plants safeguard growing apices from pathogen infection is still a mystery. The shoot apical meristem (SAM) is one of the three stem cells niches that give rise to the above ground plant organs. This is very well explored; however, how signaling networks orchestrate immune responses against pathogen infections in the SAM remains unclear. To reconstruct a transcriptional framework of the differentially expressed genes (DEGs) pertaining to various SAM cellular populations, we acquired large-scale transcriptome datasets from the public repository Gene Expression Omnibus (GEO). We identify here distinct sets of genes for various SAM cellular populations that are enriched in immune functions, such as immune defense, pathogen infection, biotic stress, and response to salicylic acid and jasmonic acid and their biosynthetic pathways in the SAM. We further linked those immune genes to their respective proteins and identify interactions among them by mapping a transcriptome-guided SAM-interactome. Furthermore, we compared stem-cells regulated transcriptome with innate immune responses in plants showing transcriptional separation among their DEGs in *Arabidopsis*. Besides unleashing a repertoire of immune-related genes in the SAM, our analysis provides a SAM-interactome that will help the community in designing functional experiments to study the specific defense dynamics of the SAM-cellular populations. Moreover, our study promotes the essence of large-scale omics data re-analysis, allowing a fresh look at the SAM-cellular transcriptome repurposing data-sets for new questions.

## 1. Introduction

Unlike animals, plants are sessile and cannot defend themselves from potential danger through the mechanism of fight and flight. Being directly exposed to a biotic and abiotic environment, they perceive plethora of signals that impact their physiological wellbeing. They often re-arrange their internal networks in response to external cues in order to adjust their developmental priorities. Moreover, they regularly replace body organs and tissues, such as leaves, flowers, and cambium, as a token of commitment to their overall *Bauplan* (body plan) [1]. In plants, the pluripotent stem cells constantly supply precursor daughter cells to form groups of cells with identical functions (differentiated tissues) that culminates in body organs [2,3,4]. Stem cell niches, such as the shoot apical meristem (SAM), the root apical meristem (RAM), and the lateral meristem, maintain a specific signaling environment that prevent stem cells to undergo differentiation all at once yet keep a definite number of meristematic stem cells in an undifferentiated state [3,4]. Being a high value target inside the plant body, the SAM signaling program must not be interfered by external biotic and abiotic factors. In nature, disease-free seeds and plants can be produced from plant apexes because stem cells of the SAMs are growing sterile despite infections in other differentiated organs of the plants [5,6,7,8]. However, the molecular mechanisms that orchestrate immunity in the SAM are largely unexplored.

In *Arabidopsis*, the stem cell niche SAM is comprised of three distinct regions, the central zone (CZ) is situated at the apex of the SAM and is the custodian of the pluripotent stem cells of the plant. The multipotent stem cells of the CZ give birth to the cells of the peripheral zone (PZ) [3]. Beneath the central and PZs, the layer of the SAM is known as the rib meristem. The cells of this zone ultimately grow into vasculature cells. To ensure the existence of the subsequent generations of a plant (via. seeds and flowers), the stem cells constantly provide precursor cells to replenish the need of recruiting cells into aerial organs, such as shoot, leaves, and flowers [3,4,9,10]. The SAM cell populations are divided into numerous cell types, having domains of various proteins that are always fused; hence, it is very challenging to differentiate a specific cell population in a distinct cell zone, such as CZ and PZ. Thus, the enrichment of individual SAM cellular populations is a real bottleneck. However, genes exclusively expressed in a given cellular population can be exploited for the enrichment of that population. The SAM cell populations that can be enriched on the basis of cell specific markers are: WUS-expressing cells, AtHB8-layer of cells, FIL, S17, CLV3p-expressing cells (stem cells), HGD-layer, LAS cell layer, KAN1, and HMG layers of the SAM cellular populations [11,12]. Once enriched, these individual cell populations can be subjected to transcriptomics and metabolomics investigation. In our meta-transcriptomics approach, we comprehensively analyze immune signatures in various SAM cell populations and catalogue genes that participate in immune function of the *Arabidopsis* SAM.

Hormonal interplay has an important role in the initiation and maintenance of the SAM. Plant hormones cytokinins (CKs) and auxin are the key small-molecule hormones that maintain the integrity stem cells in the SAM [4,9,13]. In *Arabidopsis*, increased cytokinin responses expedite the meristematic activities, whereas reduction in cytokinin signaling culminates in reduced meristem size [14,15]. The induction of *Shoot Meristemless (STM)* induces the transcription of the cytokinin biosynthetic enzyme gene *Isopentenyltransferase* 7 (*IPT7*). Moreover, *stm-1* mutants fail to initiate the SAM formation, suggesting that STM-mediated cytokinin activation plays an important role in the SAM formation [3]. Likewise, auxin inhibits the meristem promoting activities of cytokinin in the leaf primordium [4,9]. Thus, an antagonistic interaction between auxin and cytokinin plays a pivotal role in regulating the dynamics of SAM signaling networks in plants. Whether the defense-related hormones, such as jasmonic acid and salicylic acid, perform immune-related functions in the SAM is still not known.

The CVL3 (CLAVATA3)/WUS-mediated module in the SAM and the binding of CLV3p to CLV1 and CLV2 receptor complex play important roles in the maintenance of stem cell activities [4,10]. In a different context, and although still under debate, the CLV3p [10] is believed to interact with the well-known innate immune receptor Flagellin Sensing 2 (FLS2). Similar to flg22 (22-amino acid bacterial-epitope) [16] https://www.ncbi.nlm.nih.gov/pmc/articles/PMC4214217/ - B29, CLV3p has been shown to bind with FLS2 and activate Mitogen-Activating Protein Kinase (MAPK), which induces downstream immune-related genes [5,8,16,17]. The flg22 based activation of FLS2 normally results in growth inhibition of seedlings, and the CLV3p-FLS2 driven immune response in the SAM does not lead to growth penalties [6,8]. Other studies proved the contrary and demonstrated that FLS2 is completely blind to CLV3p and that any activation of FLS2 other than flg22 can be attributed to potential contamination of epitopes that are expressed in bacterial systems [18,19,20,21]. Both these findings have their own merits and demerits and thus demand further research down these lines.

The immune-related functions of the SAM are relatively less characterized as compared to other plant parts. The main objective of this study was to collect a vast array of transcriptomes that deal with various SAM cellular populations. We further aimed at subjecting them to enrichment analysis for immune functions. Another objective of this study was to investigate the transcriptional basis of CLV3p-triggered immunity in the SAM and compare it with flg22-based typical innate immune responses in plants. We further aimed at mapping a transcriptome guided SAM interactome to better comprehend immune-related modules in the SAM cellular populations. The overall purpose of this study was hence to provide a systems biology framework of the immune-related genes and their regulation in 10 different SAM cellular populations. Emphasizing the essence of large-scale data re-usability, our analysis offers repertoire of genes that can be functionally dissected to unearth the molecular basis of defense mechanisms in the SAM.

## 2. Results

### 2.1. Acquisition of the Arabidopsis Transcriptome Datasets Pertaining to SAM Cellular Populations and PAMPs-Treated Mesophyll Cells

The *Arabidopsis* SAM cellular populations are comparatively well studied for aspects of plant growth and development. However, immune responses of the various SAM cellular populations are not well studied. To reconstruct a transcriptional framework of the SAM cellular populations, we acquired 40 sets (raw data) of gene expression profiles from Gene Expression Omnibus (GEO) pertaining to various SAM cell populations, such as CLV3p-expressing stem cells belonging to the CZ, FILP-expressing cells belonging to the organ primordia/PZ, WUS-expressing cells belonging to the RZ, AtML1-expressing cell belonging to the epidermal cell layer, and AtHGD4 cells belonging to the sub-epidermal cell layer, HMG-expressing cells of epidermal cell types; likewise, KAN- and LAS-expressing cells belong to the peripheral zone cell types (see Table 1 for details). Moreover, the transcriptome of the cells belongs to vasculature cell types, such as shoot xylem cells/AtHB8 gene expressing cells and shoot phloem cells/ S17-gene-expressing cells, are also included in the analyses. To get a specific insight into the transcriptional regulation of stem cell populations (the CLV3p-expressing cells), we also included a non-functional (mutant) CLV3n-expressing cells to be compared with functional (wild type) CLV3p-expressing stem cells (Table 1, Table S1). Furthermore, we included a flg22-treated mesophyll cell gene expression profile in our study in order to find potential regulatory overlap and contrast it with CLV3p-mediated transcriptional signatures in the SAM [5]. The transcriptional profiles of all these cell populations (Table 1) were based on cell sorting and protoplasting procedures. To eliminate the effects protoplasting (treatment of the cells with cell wall degrading enzymes [12]) have on the transcription of the SAM-cellular populations and the flg22-treated mesophyll cells, we further included microarray datasets that deal with gene expression changes in cellular populations upon the application of protoplasting cocktails (Table 1).

### 2.2. Normalization and Analysis of the SAM Cellular Transcriptomes and Functional Categorization of the DEGs (Differentially Expressed Genes)

To maximally reduce batch and source effects in the analysis of the acquired SAM-based transcriptomes, we re-normalized all datasets of all 3 SAM populations (CLV3, FIL, WUS) acquired from the GEO accession GSE13596 and 7 SAM-cellular populations under the accession GSE28109. Each set was normalized and further analyzed for differential gene expression (DEGs) with R package limma. Taking each individual cellular population as test and the rest of the SAM-cellular populations as background, we identified DEGs in all 10 SAM-cellular populations (Table S1). After excluding 301 protoplasting induced genes, and based on the criteria of adjusted p-values and fold changes, we identified DEGs for SAM cell populations as follows; 192 for CLV3p-expressing cells, 43 for WUS-expressing cells, 143 for SILP-expressing cells, 85 for at ML1-expressing cells, 48 for LAS-expressing cells, 120 genes for KAN1-expressing cells, 188 genes for HMG-expressing cells, and 128 genes for HDG4 cells (Table S1). It is noteworthy to mention that the number of DEGs of AtHB8 and S17 cellular populations are manifold more than the rest of the SAM-cells. For instance, the DEGs of S17-expressing cells are 2824 and that of At HB8-expressing vasculature cells are 1862. Except for the vascular cells, most of the SAM cellular populations have distinct gene expression prolife, with minimal overlap among DEGs of the SAM cells (Table S1).

**Figure 1:**
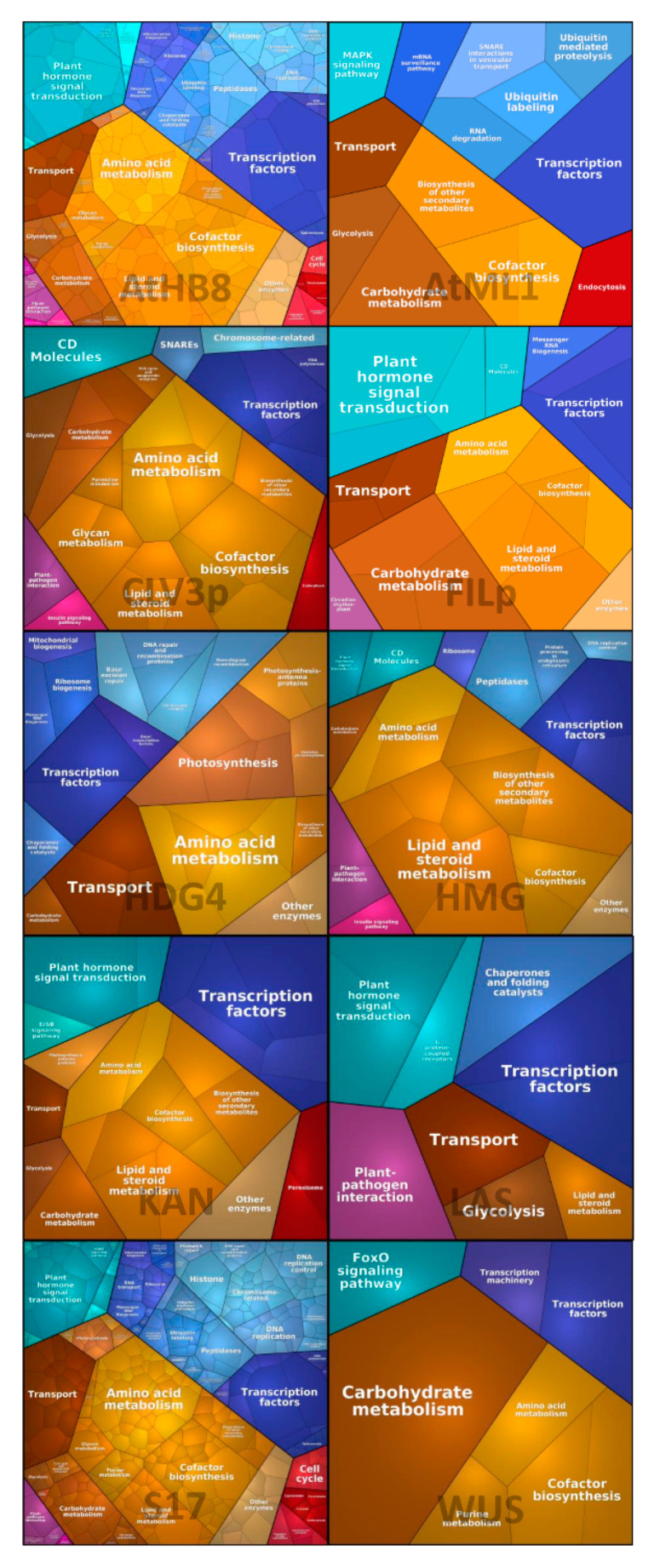
Voronoi Tree-maps for the visualization of differentially expressed genes (DEGs) of the SAM cellular populations with hierarchical classification based on KEGG pathways. The DEGs of the 10-SAM cellular populations (HGD, FILP, CLV3p, AtMIL, AtHB8; upper row: WUS, S17, LAS, KAN, and HMG: Lower row) are separately visualized via the Bionic Visualization tool (https://bionic-vis.biologie.uni-greifswald.de/). To get functional insight, and for global understanding of multiple gene expression datasets belonging to various cellular populations of the SAM, the visualization of the functional relatedness of the genes is displayed here by tessellations: Each gene is shown by a polygon and functionally related genes are arranged in common and similarly colored regions. Main functional categories (KEGG ontologies) are shown with different color codes, such as metabolism, genetic information processing, plant-pathogen interactions, and environmental information processing, as well as cellular processes, such as cell growth, mobility, and cell death.

**Figure 2:**
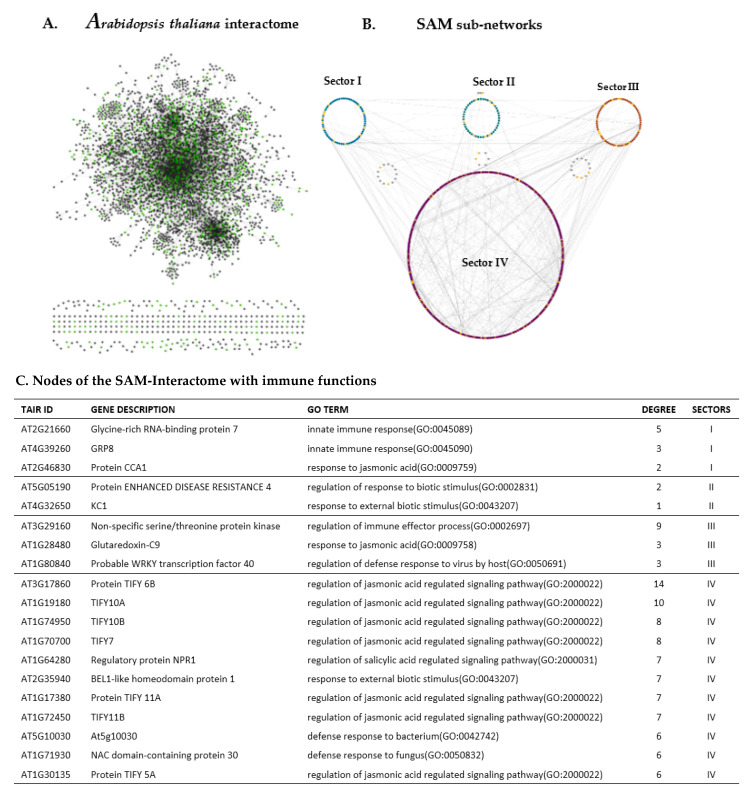
*Arabidopsis* transcriptome guided SAM immune interactome network analysis. (**A**) The *Arabidopsis thaliana* generic interactome (AT-Interactome) according to the Plant Interactome Database (http://interactome.dfci.harvard.edu/A_thaliana/index.php?page=download) is visualized in Cytoscape3.7.1. Nodes are denoted as dots, while non-directional edges are shown as gray lines between nodes. Green nodes in the network are proteins in which genes are differentially expressed in the SAM. The network with average connectivity among the nodes is clustered together (upper panel), while sparsely connected (liner nodes) in the networks are shown below the densely connected networks. (**B**) The differentially expressed genes (DEGs) from all 10 SAM populations are mapped onto the AT-interactome (nodes in green color) to contextualize the SAM protein-protein interaction network. Based on immune-enrichment analysis (GO terms pertaining immune/defense function), the nodes of the SAM-interactome with immune functions are highlighted in yellow. The SAM-interactome is further deconvoluted into four prominent sectors (Sector I: LAS-, HGD4-, and CLV3p-expressing cells, Sector II: AtML1- and HMG-expressing cells, Sector III: KAN-, FILp-, WUS-expressing SAM cells, and Sector IV: S17- and AtHB8-expressing cells). (**C**) Nodes of the SAM interactome with immune functions and their connectivity and functional annotation.

In order to document the quantitative composition of all 10 SAM-cellular transcriptomes with a focus on the function of each DEG, we used Voronoi Tree-maps to visualize cellular gene expression datasets in conjunction with hierarchical classifications based on Kyoto Encyclopedia of genes and Genomes (KEGG) ortholog. Voronoi Tree tessellations group functionally relate genes whose expression patterns are highly correlated across the different experimental conditions. As shown in Figure 1, each functional category is shown by a color-coded polygon. Broadly, the DEGs of the SAM cellular populations participate in processes, such as cellular metabolism, transcription, replication, and DNA repair mechanisms (Figure 1). Likewise, signal transduction and cell mobility, as well as immune defense-related functions are overrepresented in our analysis. Owing to the higher number of DEGs of the vascular SAM-cellular populations, the number of polygons is several folds greater in these cells in comparison to other SAM cell populations (Figure 1). Although the plotted Voronoi Tree-map represents many different functional categories, the scope of this study was limited to cataloguing immune-related transcriptional signatures in the SAM. According to our analysis, the DEGs of various SAM-cell populations, such as HMG, LAS, CLV3p, and FILP-expressing cells, participate in plant-pathogen interaction pathways. Likewise, the DEGs of vasculature SAM cell populations are also shown to have a role in plant-pathogen interaction functions (Figure 2).

### 2.3. GO-Based Enrichment Analysis of the SAM-Cellular Transcriptome for Immune-Related Functions

The Voronoi Tree-maps can efficiently visualize transcriptomes with respect to their regulated pathways, and we already found that several of the SAM cellular populations DEGs (Figure 2) participate in plant-pathogen interaction functions (Figure 2). However, we further adopted an inclusive approach to focus on SAM-based immune genes and subjected the DEGs of individual cellular populations to GO-based enrichment analysis. Based on a previous study for the SAM cellular *Arabidopsis* transcriptome, where shoot and root derived microarrays were assessed for relative closeness in term of their transcriptome profile [12], we categorized SAM cell populations into four clades (sectors); LAS-, HGD4-, and CLV3p-expressing cells as Sector I; AtML- and HMG-expressing cells as Sector II; KAN-, FIL-, and WUS-expressing SAM cells as Sector III; and S17- and HB8-expressing cells as Sector IV in the SAM region. The total list of DEGs for each class is investigated for enriched biological processes using Fisher’s exact test, no correction, and *p*-value limit of 0.05. The keywords “immune defense, pathogen infection, biotic stress, salicylic acid, and jasmonic acid” were used to collect immune-related GO terms for SAM DEGs.

According to our analysis (all immune-related DEGs passing the log2FC threshold in Table S4), 19 DEGs (Table 2) of the CLV3p-expressing (stem) cells participate in various immune functions, such as regulation of defense response by *Exocyst subunit Exo70 family protein, Cathepsin B-like protease 3*, *Beta-D-glucopyranosyl abscisate beta-glucosidase*, and *Tryptophan aminotransferase-related protein 2*. Likewise, *Lipoxygenase 3*, *chloroplastic* is a DEG of the stem cells that participates in jasmonic acid biosynthesis. Moreover, DEGs of CLV3p-expressing SAM cells that participate in innate immune responses are *E3 ubiquitin-protein ligase ATL31* and *CLAVATA 3*,as well as *NIMIN1.* Similarly, the *E3-Ubiquitin ligase PUB23* participates in immune effector functions and is expressed by CLV3p-expressing cell. The HGD4 cellular populations express nine DEGs that participate in immune functions, such as defense response to bacteria and fungi, innate immune functions, and jasmonate biosynthesis, as well as defense response to other organisms (Table 2, details in Table S4). Similarly, three of the LAS cells DEGs are enriched in GO terms, such as defense to fungi and defense to bacteria, as well as the jasmonate biosynthesis.

We further analyzed the DEGs that belong to SAM-AtML1- and HMG-expressing cellular populations for immune-related process (Table 3, details in Table S4) and found that five of the AtML1 cells express genes, such as *Protein PAT1 homolog*, *Duplicated homeodomain-like superfamily protein*, *Syntaxin-43*, *TIFY domain/Divergent CCT motif family protein*, and *Porphobilinogen deaminase*, participate in immune-related functions. These DEGs are enriched in GO terms, such as innate immune response, response to external biotic stimulus, and regulation of defense response, as well as response to jasmonic acid (Table 3, details in Table S4). Likewise, the SAM-HMG-expressing cellular population expresses 25 genes which are enriched in immune functions, such as response to external biotic stimulus, response to jasmonic acid, regulation of defense response, defense response to bacterium, regulation of innate immune response, defense response to fungi, and defense response to other organisms (Table 3, details in Table S4). Prominent genes regulating these processes are: *Chitinase family protein*, *Linoleate 9S-lipoxygenase 1*, *Receptor-like protein 12*, *WRKY transcription factor 17*, *Leucine-rich repeat (LRR) family protein*, *Pathogenesis-related thaumatin superfamily protein*, *Enhanced Disease Resistance 4 M(EDS4)*, *Respiratory burst oxidase homolog protein D*, *DCD (Development and Cell Death) domain protein*, and so forth (Table 3).

The third clade of the SAM cellular populations is comprised of FIL, KAN1, and WUS expressing cells. As per our analysis, the FIL-expressing SAM cellular population express 23 DEGs that participate in diverse immune processes, such as regulation of immune system processes, defense response to bacteria, and fungi as well as other organisms (Table 4, details in Table S4). Likewise, response to jasmonic acids and jasmonic acid biosynthetic process are among the overrepresented functions. *NIMIN1*, *4-coumarate--CoA ligase-like 5*, *Glutaredoxin-C9*, *Pathogenesis-related thaumatin superfamily protein*, *pectinesterase inhibitor 12*, *Serine/threonine-protein kinase OXI1*, and *WRKY transcription factor 48*, as well as *NPR5* and *NPR6*, are among the genes that may regulate immune response in FIL-expressing cells in the SAM of *Arabidopsis* (Table 4, details in Table S4). The KAN1-expressing cellular population regulate the expression of 15 genes that also participate in immune functions. *Pathogenesis-related thaumatin superfamily protein*, *Defensin-like protein 13*, *CWINV1*, *Lipoxygenase 2*, *chloroplastic*, *Beta carbonic anhydrase 1*, *chloroplastic*, *L-type lectin-domain containing receptor kinase IV.3*, and *Powdery mildew resistance protein containing protein* are DEGs that regulate defense to fungi and defense to bacteria, as well as response to external biotic stimuli (Table 3, Table S4). As per our analysis, the WUS-expressing cells of the SAM only regulate 5 genes that participate in immune-related processes, such as regulation of the immune effector process, defense response to biotic stimuli, and defense response to fungi (Table 4, details in Table S4). The fourth clade of the SAM cellular populations is comprised of S17- and HB8-expressing cells populations. Being in a more differentiated zone (vasculature), they own many times more DEGs than their counterpart in the central zone. They have many genes that participate in immune functions (Table S2). Overall, there are many genes in the SAM cellular populations that participate in immune functions and are differentially regulated without the SAM being exposed to pathogen infections.

### 2.4. Transcriptome Guided SAM Interactome and Insights Into the Proteins of Immune-Related DEGs of the SAM-Cellular Populations

As mentioned above, the SAM is the niche of stem cells population (CLV3p-expressing cells) in the central zone that gives rise to cells of the peripheral zone which turn into differentiated cells of organ primordia. The cells of the various SAM layers (Reference [11,12]: Table 1) share boundaries through transmembrane proteins or cells of one layer may diffuse into another layer/s. We mapped an interactome (protein-protein interactions: acquired from *Arabidopsis* Interactome Database) by taking the DEGs of all 10-SAM cellular populations into account. Cellular interactomes are usually generic; however, proteome or transcriptome datasets can be used to define a specific context in a general interactome. We visualized the *Arabidopsis* Interactome by using the system biology tool “cytoscape” and mapped SAM-transcriptomes (Figure S1) onto the generic *Arabidopsis* Interactome (Figure 2A: left panel). We then extracted the transcriptome guided SAM interactome out of the generic interactome and further mapped it into four sub-sectoral networks (Figure 2A: right panel). Each sub-network (sectors) represents cellular populations based upon their closeness [12] in terms of their transcriptional behavior (Table 2, Table 3 and Table 4; Sector I: LAS-, HGD4-, and CLV3p-expressing cells; Sector II: AtML- and HMG-expressing cells; Sector III: KAN-, FIL-, and WUS-expressing SAM cell; and Sector IV: S17- and HB8-expressing cells). We analyzed the SAM-Interactome to get further insight into the network behavior of immune-enriched genes among SAM-cellular populations.

As per our network analysis, Sector IV has high intra-sectoral interactions, which is contrary to the intra-sectoral behaviors of the nodes of Sector I, II, and III. However, the nodes of all fours sectors are engaged in high intersectoral interactions (Figure 2A: right panel). It is noteworthy to mention that the first three sectors (Sector I, II, and III) are engaged to a higher degree of interaction with nodes of Sector IV in comparison to the interactions within the first three sectors. We further analyzed the network behavior of proteins, the genes of which are enriched in immune functions (Table 2, Table 3 and Table 4) and are also differentially expressed in the SAM cellular populations (Table S1). We used the cytoscape plugin network-analyzer and found the degree of interaction for densely connected immune nodes (hubs) of the network (Figure 2B). As per our analysis, only three nodes of Sector I have a degree more than or equal to two, Glycine-rich RNA-binding Protein 7 interacts with 5 other proteins in the network. Likewise, GRP8 and CCA1 have three and two interacting partners in SAM-interactome, respectively. The proteins of the immune genes of the Sector II are sparsely connected and only one of their proteins, Enhanced Disease Resistance 4 (EDS4), has just two interacting partners. Contrary to Sector II, the immune proteins of Sector III are densely connected. For instance, non-specific serine/threonine protein kinase has 9 interacting partners in the SAM-interactomes, whereas Glutaredoxin-C9 and WRKY 40 each have three interactors (Figure 2B). The immune proteins of Sector IV are highly connected and have degree ranges from 6 up to 14 for some of the proteins. Besides degree of the immune-related proteins of the SAM-interactome, we also analyzed the topological behavior of the SAM-Interactome and found features of a typical regulatory network in our transcriptome guided SAM-Interactome network (Figure S1).

### 2.5. Transcriptional Basis of the Arabidopsis CLV3p-Triggered Genes in the SAM and its Comparison with flg22-Mediated Gene Expression in the Mesophyll Cells

**Figure 3:**
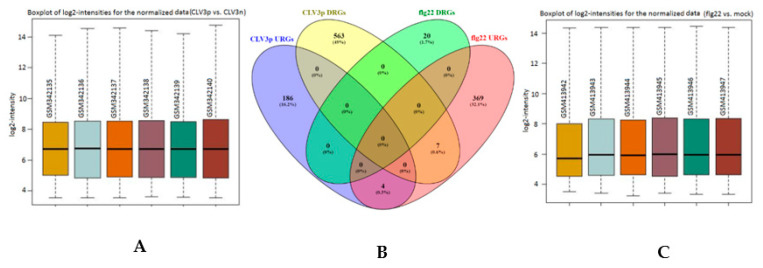

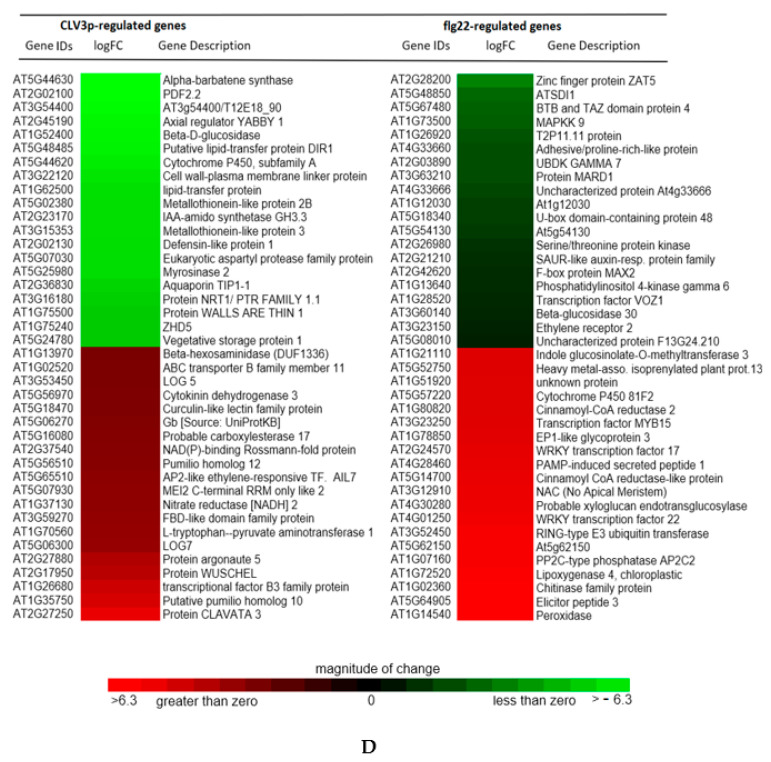
Overlap between the DEGs of CVL3p-expressing cellular populations (stem cells) and flg22-treated mesophyll cells. (**A**) The GEO acquired raw CLV3p- and CLV3n-expressing SAM cellular microarray datasets, 3-replicates each wild type and mutant with its GEO-Samples (GSM)-identifiers, are statistically normalized. (**B**) The Flg22 and CLV3p regulated differentially expressed genes (DEGs) are compared and contrasted for co-regulated genes in a Venn diagram (http://bioinfogp.cnb.csic.es/tools/venny/). (**C**) The raw microarray datasets pertaining the flag22-treated mesophyll cells and mock-treated mesophyll cells were also acquired from GEO and then normalized. (**D**) Top-20 up- and down-regulated differentially expressed genes from both CLV3p-expressing SAM cell populations (A: left panel) and flg22-treated mesophyll cells (C: right panel) are visualized with the Heatmapper plus tool (http://bar.utoronto.ca/ntools/cgi-bin/ntools_heatmapper_plus.cgi) of the ePlant database. The column information deals with the gene IDs, logFC in term of heatmap, and the annotation of the given genes. The scale bar beneath the columns denotes the magnitude of both up-regulation (red) and down-regulation (green) for the given genes.

There is only one report in the literature that attributes the naturally immunized status of the SAM to CLV3p-FLS2 mediated immunity [8]. It is generally believed that FLS2, in its interaction, is only sensitive to flg22, which is an epitope of the bacterial protein flagellin and has been widely used in studies of innate immunity in plants. To compare the transcriptional basis of CLV3p-mediated transcriptional regulation in the SAM and flg22-regulated genes in a differentiated mesophyll cells, we retrieved transcriptome profiles of “CLV3p vs CLV3n” of the SAM-stem cell population and “flg22 vs Mock” of the *Arabidopsis* mesophyll cells. We split the DEGs regulated by both conditions into Up-Regulated Genes (URGs) and Down-Regulated Genes (DRGs). In order to identify a transcriptional overlap between these two conditions, we plotted the DEGs of both experiments in a Venn diagram (Figure 3). In our analysis, we did not find any reasonable overlap between the regulated genes of these distinct cellular populations. Four genes which are up-regulated by CLV3p are also up-regulated by flg22, whereas no overlap was observed among the down regulated genes of both conditions (Figure 3). However, seven genes which are up-regulated by flg22 in mesophyll cells are shown to be down-regulated by the CLV3p in the SAM-stem cells population. Among the CLV3p and flg22 four mutually upregulated genes, two of them are cysteine/rich transmembrane domain proteins, while the other two belong to the dehalogenase and cytochrome p450 protein families (Table S3). We did not find mutually downregulated genes caused by the peptides flg22 and CLV3p. The genes which are reciprocally regulated in these two conditions belong to; protein families of sucrose transporters, LOGs, and E3-Ligase, genes related to the maintenance of the shoot apical meristem, toxin extrusion, and nitrogen metabolism (Table S3). Apparently, these genes have no direct involvement in the immune processes. We further analyzed the top 20 up and down differentially regulated genes by both flg22 and CLV3p in their respective cell types. Although there is no overlap among the top 20 up/down regulated genes mediated by both these proteinaceous ligands (flg22 and CLV3p), there is a sharp contrast in the magnitude of their regulation; the repression on gene expression that is caused by flg22 is quite mild in comparison to the stark reduction imposed by CLV3p on the SAM stem cell genes. Likewise, the induction of flg22 is higher than CLV3p in up-regulating genes in their respective cell types (Figure 3). The CLV3p up-regulated DEGs are mostly involved in SAM signaling networks, such as *CLV3p*, *WUS*, *LOGs*, and *CKX*. In contrast, the up-regulated DEGs of flg22 are typical immune-related genes, such as *WRKY 17* and *WRKY22*, *Peroxidase*, *lipoxygenase* and *Chitinase family proteins*, *Elicitors* and *PAMP-induced peptides,* and so forth (Figure 3: lower panel). From these results, we conclude that, although CLV3p and flg22 (two different peptides in terms of sequence and structure) interact with the very same plant innate immunity receptor (FLS2), their transcriptional outputs are very different.

## 3. Discussion

The sterile nature of plant apexes is exploited to produce disease-free plants, which is an old horticultural practice. The exact mechanisms why plant tips grow aseptic is still a *terra incognita* for the field of plant immunity. The *Arabidopsis* SAM stem cell niche is an aggregation of approximately 500 cells located at the growing tip of each shoot [9,11,12]. The CZ of the SAM harbors stem cells, the progeny of which are displaced into the adjacent PZ, where they proliferate before differentiating. Despite much progress in understanding the dynamics of immune responses of plants in general, the defense mechanism of the SAM stem cell niche is hardly assessed [5,6,7,8]. The compact nature of cellular populations in the form of layers in the SAM makes it less amenable to perform typical plant-pathogen interaction experiments specifically in the niche where stem cells reside. However, the advent of single cell genomics, advanced microscopy, and sorting techniques, together with sequencing technologies, provide ease in assessing the SAM and RAM cellular populations for detailed analysis. Profiting from these technological advancements, the dynamics of growth and development regulated by stem cells activity in these niches are intensively investigated these days.

Giving momentum to the essence of large-scale data re-usability, as well as re-producibility, we applied a systems-based deconvolution approach to catalogue immune-related genes belonging to various SAM-cellular populations. We acquired raw data pertaining the transcriptomes of 10-SAM cellular populations [12] from the public repository GEO and assessed DEGs (Figure S1) testing each population against all other cellular populations. Besides finding DEGs across various experimental conditions (cell types and cell treatments), we applied double enrichment analysis: Voronoi Tree-maps as general visualization of DEGs based on KEGG pathways (Figure 1) and GO-based enrichment of the DEGs of the SAM cellular populations for immune functions (Table 2, Table 3 and Table 4). In a previous study, Yadav et al. [12] compared DEGs in terms of specific cell populations as group to see the impact of outliers in distinct cell layers (L1, L2, and L3). However, we are broadly interested in genes contained in the SAM cellular populations so as to document their immune functions and motivate experimentalists to further decipher immune functions applying functional genomics approaches.

SAM cellular populations follow a conservative strategy by regulating only a limited number of genes that address their functional requirements. Being committed to functions of meristematic activities, their specific gene expression profile is way too small in comparison to the DEGs in differentiated tissues; the latter express 1000s of genes under given circumstances. We found the response to fungi, response to bacterium, and response to jasmonic acid and jasmonate biosynthesis as statistically overrepresented immune functions in the SAM-cellular populations. CLV3p, HMG, and FILP-expressing cells are among the SAM cellular populations that express immune-related genes (Table 2, Table 3 and Table 4). Both CLV3p [5] and HMG [12] cells have previously been shown to express genes that are implicated in pathogen defense. Likewise, the cells of S17 and HB8 populations express several genes that contribute to immunity against pathogen infection in plants. As compared to other SAM cell types, we found (Table S2) that 50% of the DEGs are co-regulated by both S17- and HB8- expressing cells of the SAM. Looking into the number of genes that are enriched in immune functions of the SAM, we conclude that, without being exposed to a pathogen, SAM already has a pre-meditated immunity, whereby various layers (cell populations) express immune genes as a preemptive measure in safeguarding the integrity of the SAM.

To get deeper insight into the function of immune-enriched genes, we linked SAM-DEGs to their encoded proteins. We contextualized a generic interactome AI (*Arabidopsis* Interactome) into SAM-interactome by mapping DEGs of all SAM-cellular populations onto the AI. The four defined sectors (Figure 2A) of the SAM-interactome have individual cellular populations that share similarities in their gene expression profile [12]. Nodes of the sub-networks have poor intra-sectoral interactions (edges), as well as sparse intersectoral interaction, among the nodes of Sector I, II, and III have been observed (Figure 2). However, the vast majority of the cross-sectoral interactions happen between the nodes of Sector IV and first three sectors.

There exists a reasonable skepticism about the validity of high-throughput Y2-H based interactomes owing to false positive/negative interactions and technological discrepancies in detecting interactions between soluble proteins and insoluble ones in the cell. Despite this valid skepticism, the computational approaches applied on large cellular interactomes in detecting functional modules endow the experimentalists with a handle to dissect the biological complexity with more targeted approaches. A pertinent question concerning the mapped SAM-interactome (Figure 2) is: How do interactions between proteins of different cellular populations interact within the SAM’s sectors and layers? It remains to be established how proteins of various cellular populations and sectors can interact as a network inside the SAM. There are three plausible explanations to address this apprehension: the movement of the cellular populations inside the SAM; the movements of the proteins, such as WUS, CLV3p across the cells; and the occurrence of transmembrane protein domains at the cellular interfaces in the SAM. Under these circumstances, proteins of different cells may interact as a network in the SAM despite belonging to different cellular population. However, there might be other modes of protein-protein interactions in the SAM-cellular populations, and that margin of error in interactions (artefacts of methods that generate interactomes) cannot be ruled out completely. We identified hub nodes in the interactomes which participate in defense functions and have sectoral and cross-sectoral interactions in the SAM-Interactome. These hub nodes (Figure 2) merit further investigation to fully unleash their role in SAM-mediated immunity in plants.

An interesting mechanism of bacterial cleansing in the SAM was demonstrated to involve the binding of CLV3p (stem cells expressed gene) to FLS2 and the subsequent activation of Mitogen-Activating Protein Kinase (MAPK) that culminates in the expression of PAMP-Triggered-Immunity (PTI) marker genes in *Arabidopsis* [5,17]. Contrary to flg22-FLS2 mediated immunity, which involves the inhibition of seedlings growth, the CLV3p-FLS2-triggered immunity operates without growth penalties [6]. This mechanism of stem-cell-triggered immunity was refuted by other reports, demonstrating that CLV3p is blind to FLS2 in eliciting immune responses and that CLV3p does not induce the expression of innate immunity genes via the receptor FLS2 [18,19]. Taking these reports into account, we compared a large scale-transcriptional response mediated by flg22 and CLV3p in their respective cellular populations (Figure 3). Our results clearly demonstrate that there is no reasonable overlap in the DEGs of CLV3p-expressing stem cells population and flg22-treated mesophyll cells. Despite binding the same receptor, the structurally different peptides flg22 and CLV3p regulate different sets of genes in *Arabidopsis.*

In conclusion, we deconvoluted a set of immune-related genes from large-scale transcriptome data belonging to different SAM-cell populations. The identified transcriptional framework will help the plant research community in dissecting mechanisms of defense in the SAM. Our systems biology analysis infers that not only a single cell population of the SAM is committed to immune functions; rather, many cell types, in their respective layers, express genes that potentially participate in immune processes in the SAM. Once the mechanism of SAM-mediated immunity is fully elucidated, this will provide unique opportunities to incorporate pathogen resistance conferring pathways in crop plants. One of the candidate genes that can create impact in a breeding program is CLV3p, the expression of which is more restricted to the stem cell zone of the SAM. Our systems biology analysis suggests that CLV3p-expressing cells also induce many defense-related genes, which are different from those induced by the flg22-triggered-FLS2-mediated pathway in plants. Whereas the activation of FLS2 based immune pathway has a growth penalty, the CLV3p mediated immunity does not inhibit plant growth and has already been tested in *Arabidopsis* [6,7]. Thus, the incorporation of such a mechanism in crop plants will have dual benefits: breeding for pathogen resistance and no growth arrest due to active defense responses. It is noteworthy to mention that the introduction of stem cell specific pathways in differentiated plant tissues might have pleiotropic consequences. Therefore, expressing such pathways under inducible promoters would offer a need-dependent expression of the transgene that might reduce the off-target effects of the transgene which sometimes prove more deleterious than the anticipated positive effects on yield and protection. In order to fully translate the benefits of stem cell triggered immunity in crop plants, it is direly required to first establish assays which should easily allow plant-pathogen interaction experiments in the SAM of various crop plants. However, the modern genome editing techniques, state-of-the-art microscopy and imaging analysis, as well the non-invasive plant phenotyping approaches, should allow us to capitalize on the translational benefits of SAM-triggered immunity in crop plants.

## 4. Material and Methods

### 4.1. Source of Transcriptomes (Gene Expression Data/Profiles): Gene Expression Omnibus (GEO)

We acquired microarrays datasets with accession numbers GSE13596, GSE16472, and GSE28109 from GEO, which is a public depository of transcriptomics/genomics studies obtained with microarrays and sequence-based experiments. The database provides the data, along with the publication if available, and description of the study and samples (all metadata describing the experiments). The data can be directly downloaded and analyzed independently or with the help of tools provided by the database [23,24].

Specifically, GSE13596 and GSE28109 provide gene expression profiles of *A.thaliana* shoot apical stem cell populations [11] and shoot apical meristem [12], respectively; GSE16472 contains, instead, the gene expression data for the response of *Arabidopsis* mesophyll cells after bacterial flagellin (flg22) treatment. Raw data from Affymetrix *Arabidopsis* ATH1 Genome Array is downloaded as CEL files from aforementioned GEO studies. Information on samples in each data can be found in Table 1. The collected data is arranged into three different sets for further analysis. The first set is composed of 10 SAM populations with 3 populations (CLV3, FIL, WUS) being derived by the analysis of GSE13596 and 7 populations from GSE28109. The second set comprises the gene expression data of CLV3p-expressing and lacking stem cells in GSE13596 and the third set is built with the samples from GSE28109 comparing flg22-treated mesophyll cells in comparison to the mock.

### 4.2. R Packages

We normalized the acquired microarray datasets with Limma, which is a Bioconductor tool widely used for the analysis of gene expression data. It compares different conditions provided as experiment design and employs linear models to determine differential expression [25]. We also used Oligo, which is also a Bioconductor tool for the preprocessing and visualization of the oligonucleotide microarrays from Affymetrix and NimbleGen genes. It is composed of various algorithms, e.g., RMA, being commonly used as a tool for background correction and normalization of the microarray data [26,27,28,29].

### 4.3. Voronoi Tree-Maps

To visualize complex microarray datasets, we used Voronoi Tree-maps for the investigation of functional categories with the use of KEGG pathways and for visualization of functions prevailing in a given transcriptome with corresponding quantitative data. The resulting transcriptome map is a composition of polygons of different sizes and colors that represent the functional categories representing highly expressed genes in the submitted data [30].

### 4.4. The Gene Ontology Resource

To assess the analyzed DEGs for immune functions, we profited from the Gene Ontology resource, which is a comprehensive database of gene functions for diverse set of organisms. It provides “gene ontologies” annotated in terms of molecular function, biological process, and cellular component. Further analysis of gene ontologies, e.g., GO enrichment analysis, in a given gene list can be also performed using the database [31,32,33].

### 4.5. CCSB Interactome Database

The Interactome database from the Center for Cancer Systems Biology (CCSB) (http://interactome.dfci.harvard.edu/) includes interactomes from *Homo sapiens*, *Arabidopsis thaliana*, *Saccharomyces cerevisiae*, and *Caenorhabditis elegans.* We acquired protein-protein interaction datasets from CCSB and mapped transcriptomes datasets to *Arabidopsis* interactome in order to create a transcriptomes-guided SM-interactome.

### 4.6. Cytoscape 3.7.1 and NetworkAnalyzer

We used Cytoscape, which is a network visualization and analysis tool that allows the assessment of protein-protein interaction networks and signaling pathways in its core and further allows such networks for other analysis owing to several dedicated plugins it provides [34]. We used NetworkAnalyzer for analyzing SAM-interactomes in term of topological and functional analysis. It generates graphs of different topological parameters, such as node degree, number of neighbors, path length, shortest path length, and number of connected nodes [35].

### 4.7. Data Preprocessing, Analysis, and Filtering

Background correction and normalization of each set is performed separately using the RMA algorithm from the R package “oligo” [26,27,28,29]. A soft intensity-based filtering is applied on the normalized data: lowly expressed genes are filtered out by a threshold of 4 for the median intensities of genes as per the instructions of Klaus et al. [36]. Differential gene expression analysis is performed with R package limma. For each set, contrasts are created (control vs. test), and samples are annotated accordingly. After acquiring a linear model fit for each set, empirical Bayes statistics are implemented to get differential expressed genes (DEGs). The differential expressed genes are filtered against *p*-value < 0.05 and |log2FC| >1. For transcriptome sets, 301 genes reflecting the protoplasting effect are subtracted [11] from the SAM-cellular population gene expression profile.

### 4.8. Pathway Enrichment Analysis

Functional categories for differentially expressed genes in each SAM population is performed employing Proteomaps [30]. For each population, DEGs and the log2FC values are used to generate the Voronoi Tree-maps and third level functional categories that correspond to individual pathway maps in line with KEGG hierarchy levels [37], which were chosen for representation of the differentially expressed genes.

### 4.9. Gene Ontology Enrichment Analysis for Immune-Related Processes

For GO enrichment analysis, SAM populations are separated into two classes. Class I includes 8 SAM populations (FIL, WUS, CLV3, KAN, LAS1, HMG, HDG4, and ATML1: further divided into three categories based on closeness in their expression profile [12]) and class II includes 2 populations (ATHB8 and S17). The total list of DEGs for each class is investigated for enriched biological processes. Fisher’s exact test, no correction, and p-value limit of 0.05 is used for the enrichment analysis. The complete list of enriched biological processes is then checked for the keywords “immune”, “defense”, “pathogen”, “biotic”, “salicylic acid”, and “jasmonic acid”. The terms resulted from the search are collected as immune-related GO terms, and the SAM DEGs that take part in them are listed for each GO term.

### 4.10. Network Construction, Visualization, and Analysis

The *Arabidopsis thaliana* interactome is downloaded from CCSB Plant Interactome Database (http://interactome.dfci.harvard.edu/A_thaliana/index.php?page=download) and visualized in cytoscape 3.7.1 [34]. The DEGs from all 10 SAM populations are gathered together and then searched in the *A.thaliana* interactome. Selected nodes and their edges are used to construct the SAM interactome. The resulting network is analyzed for basic network properties using cytoscape network-analyzer [35]. Topological properties, including number of neighbors, path length, closeness centrality, betweenness centrality, degree, and average neighborhood connectivity, are visualized in graphs provided by the application. Similarly, immune-related DEGs listed in the previous step are searched in the SAM interactome. The detected nodes are then highlighted for further analysis.

## Figures and Tables

**Table 1:** GEO (Gene Expression Omnibus) identifiers of the transcriptomes belonging to the various shoot apical meristem (SAM) cell populations, protoplasting effects, and the source of flg22-treated mesophyll cell microarrays.

Geo Series	Geo Sample	Cell Population	Cell Type	Source [Reference]
GSE13596	GSM342135	CLV3n	CLV3n cells lacks CLV3p cell type.	Yadav RK et al. 2009 [11]
GSE13596	GSM342136	CLV3n	CLV3n cells lacks CLV3p cell type.	Yadav RK et al. 2009 [11]
GSE13596	GSM342137	CLV3n	CLV3n cells lacks CLV3p cell type.	Yadav RK et al. 2009 [11]
GSE13596	GSM342138	CLV3p	central zone/stem cells	Yadav RK et al. 2009 [11]
GSE13596	GSM342139	CLV3p	central zone/stem cells	Yadav RK et al. 2009 [11]
GSE13596	GSM342140	CLV3p	central zone/stem cells	Yadav RK et al. 2009 [11]
GSE13596	GSM342141	FILp	organ primordia/peripheral zone cell type	Yadav RK et al. 2009 [11]
GSE13596	GSM342142	FILp	organ primordia/peripheral zone cell type	Yadav RK et al. 2009 [11]
GSE13596	GSM342143	FILp	organ primordia/peripheral zone cell type	Yadav RK et al. 2009 [11]
GSE13596	GSM342144	Prot_Ind	protoplast induced SAM	Yadav RK et al. 2009 [11]
GSE13596	GSM342145	Prot_Ind	protoplast induced SAM	Yadav RK et al. 2009 [11]
GSE13596	GSM342146	Prot_Unind	uninduced SAM	Yadav RK et al. 2009 [11]
GSE13596	GSM342147	Prot_Unind	uninduced SAM	Yadav RK et al. 2009 [11]
GSE13596	GSM342148	WUSp	L3 layer/rib zone cell type	Yadav RK et al. 2009 [11]
GSE13596	GSM342149	WUSp	L3 layer/rib zone cell type	Yadav RK et al. 2009 [11]
GSE16472	GSM413942	flg22cont	treated with mock solution	Boudsocq M et al. 2010 [22]
GSE16472	GSM413943	flg22	treated with flg22	Boudsocq M et al. 2010 [22]
GSE16472	GSM413944	flg22	treated with flg22	Boudsocq M et al. 2010 [22]
GSE16472	GSM413945	flg22cont	treated with mock solution	Boudsocq M et al. 2010 [22]
GSE16472	GSM413946	flg22	treated with flg22	Boudsocq M et al. 2010 [22]
GSE16472	GSM413947	flg22	treated with flg22	Boudsocq M et al. 2010 [22]
GSE28109	GSM706470	AtML1	ubiquitous L1 layer/epidermal cell type	Yadav RK et al. 2014 [12]
GSE28109	GSM706471	AtML1	ubiquitous L1 layer/epidermal cell type	Yadav RK et al. 2014 [12]
GSE28109	GSM706472	AtHB8	shoot xylem/vasculature cell type	Yadav RK et al. 2014 [12]
GSE28109	GSM706473	AtHB8	shoot xylem/vasculature cell type	Yadav RK et al. 2014 [12]
GSE28109	GSM706474	AtHB8	shoot xylem/vasculature cell type	Yadav RK et al. 2014 [12]
GSE28109	GSM706475	HDG4	L2 layer/subepidermal cell type	Yadav RK et al. 2014 [12]
GSE28109	GSM706476	HDG4	L2 layer/subepidermal cell type	Yadav RK et al. 2014 [12]
GSE28109	GSM706477	HDG4	L2 layer/subepidermal cell type	Yadav RK et al. 2014 [12]
GSE28109	GSM706478	HMG	meristematic L1 layer/epidermal cell type	Yadav RK et al. 2014 [12]
GSE28109	GSM706479	HMG	meristematic L1 layer/epidermal cell type	Yadav RK et al. 2014 [12]
GSE28109	GSM706480	HMG	meristematic L1 layer/epidermal cell type	Yadav RK et al. 2014 [12]
GSE28109	GSM706481	KAN1	Abaxial organ boundaries/peripheral zone cell type	Yadav RK et al. 2014 [12]
GSE28109	GSM706482	KAN1	Abaxial organ boundaries/peripheral zone cell type	Yadav RK et al. 2014 [12]
GSE28109	GSM706483	KAN1	Abaxial organ boundaries/peripheral zone cell type	Yadav RK et al. 2014 [12]
GSE28109	GSM706484	LAS	Adaxial organ boundaries/peripheral zone cell type	Yadav RK et al. 2014 [12]
GSE28109	GSM706485	LAS	Adaxial organ boundaries/peripheral zone cell type	Yadav RK et al. 2014 [12]
GSE28109	GSM706486	S17	shoot phloem/vasculature cell type	Yadav RK et al. 2014 [12]
GSE28109	GSM706487	S17	shoot phloem/vasculature cell type	Yadav RK et al. 2014 [12]
GSE28109	GSM706488	S17	shoot phloem/vasculature cell type	Yadav RK et al. 2014 [12]

**Table 2:** Immune defense functions of DEGs of Sector I, the CLV3p, HDG4, and LAS cellular populations.

Gene ID	Gene Description	Go Term	Cell Population
AT1G02450	*NIMIN1*	regulation of immune system(GO:0002682)	CLV3p
AT1G07000	*Exocyst subunit Exo70 family protein*	regulation of defense response(GO:0031347)	CLV3p
AT1G37130	*Nitrate reductase [NADH] 2*	response to biotic stimulus(GO:0043207)	CLV3p
AT1G52400	*Beta-D-glucopyranosyl abscisate beta-glucosidase*	defense response to fungus(GO:0050832)	CLV3p
AT2G27250	*CLAVATA 3*	innate immune response(GO:0045087)	CLV3p
AT2G35930	*E3 ubiquitin-protein ligase PUB23*	immune effector process(GO:0002252)	CLV3p
AT3G44300	*NIT2*	response to biotic stimulus(GO:0043207)	CLV3p
AT4G01610	*Cathepsin B-like protease 3*	defense response(GO:0006952)	CLV3p
AT4G24670	*Tryptophan aminotransferase-related protein 2*	defense response to bacterium(GO:0042742)	CLV3p
AT5G27420	*E3 ubiquitin-protein ligase ATL31*	innate immune response(GO:0045087)	CLV3p
AT1G31280	*Protein argonaute 2*	defense response to bacterium(GO:0042742)	HDG4
AT1G74930	*Ethylene-responsive transcription factor ERF018*	defense response to organism(GO:0098542)	HDG4
AT2G21660	*Glycine-rich RNA-binding protein 7*	innate immune response(GO:0045087)	HDG4
AT4G16950	*Disease resistance protein RPP5*	defense response to fungus(GO:0050832)	HDG4
AT4G34710	*Arginine decarboxylase 2*	response to jasmonic acid(GO:0009753)	HDG4
AT4G39260	*GRP8*	innate immune response(GO:0045087)	HDG4
AT5G15380	*DNA (cytosine-5)-methyltransferase DRM1*	defense response to fungus(GO:0050832)	HDG4
AT5G65710	*LRR receptor-like serine/threonine-protein kinase*	defense response to bacterium(GO:0042742)	HDG4
AT5G66570	*Oxygen-evolving enhancer protein 1-1, chloroplastic*	defense response to bacterium(GO:0042742)	HDG4
AT1G11310	*MLO-like protein 2*	defense response to fungus(GO:0050832)	LAS
AT2G30750	*Cytochrome P450 71A12*	defense response to bacterium(GO:0042742)	LAS
AT2G46830	*Protein CCA1*	response to jasmonic acid(GO:0009753)	LAS
AT5G51630	*Disease resistance protein (TIR-NBS-LRR class)*	defense response(GO:0006952)	LAS

**Table 3:** Immune defense functions of DEGs of AtML1 and HMG cellular populations.

Gene ID	Gene Description	Go Term	Cell Population
AT1G79090	*Protein PAT1 homolog*	innate immune response(GO:0045087)	AtML1
AT2G36890	*homeodomain-like superfamily protein*	response to jasmonic acid(GO:0009753)	AtML1
AT3G05710	*Syntaxin-43*	regulation of defense response(GO:0031347)	AtML1
AT4G14720	*TIFY domain/Divergent CCT motif family protein*	regulation of defense response(GO:0031347)	AtML1
AT5G08280	*Porphobilinogen deaminase, chloroplastic*	response to biotic stimulus(GO:0043207)	AtML1
AT1G02360	*Chitinase family protein*	response to biotic stimulus(GO:0043207)	HMG
AT1G55020	*Linoleate 9S-lipoxygenase 1*	response to jasmonic acid(GO:0009753)	HMG
AT1G71400	*Receptor-like protein 12*	defense response(GO:0006952)	HMG
AT2G24570	*WRKY transcription factor 17*	defense response to bacterium(GO:0042742)	HMG
AT2G26380	*Leucine-rich repeat (LRR) family protein*	defense response(GO:0006952)	HMG
AT2G28790	*Pathogenesis-related thaumatin superfamily protein*	response to biotic stimulus(GO:0043207)	HMG
AT4G32650	*KC1*	response to biotic stimulus(GO:0043207)	HMG
AT5G05190	*Protein ENHANCED DISEASE RESISTANCE 4*	response to biotic stimulus(GO:0002831)	HMG
AT5G23820	*MD-2-related lipid-recognition protein 3*	defense response(GO:0006952)	HMG
AT5G47910	*Respiratory burst oxidase homolog protein D*	defense response to fungus(GO:0050832)	HMG

**Table 4:** Immune defense functions of DEGs belonging to FILp, KAN1, and WUS cellular populations.

Gene ID	Gene Description	Go Term	Cell Population
AT1G02450	*NIMIN1*	regulation of immune process(GO:0002682)	FILp
AT1G20510	*4-coumarate--CoA ligase-like 5*	jasmonic acid biosynthetic process(GO:0009695)	FILp
AT1G28480	*Glutaredoxin-C9*	response to jasmonic acid(GO:0009753)	FILp
AT1G73620	*Pathogenesis-related thaumatin superfamily protein*	response to biotic stimulus(GO:0043207)	FILp
AT2G26440	*Probable pectinesterase/pectinesterase inhibitor 12*	response to biotic stimulus(GO:0043207)	FILp
AT2G41370	*Regulatory protein NPR5*	JA mediated signaling pathway(GO:0009864)	FILp
AT3G25250	*Serine/threonine-protein kinase OXI1*	defense response(GO:0006952)	FILp
AT3G57130	*Regulatory protein NPR6*	jasmonic acid mediated signaling (GO:0009864)	FILp
AT5G48485	*Putative lipid-transfer protein DIR1*	innate immune response(GO:0045087)	FILp
AT5G49520	*Probable WRKY transcription factor 48*	defense response to bacterium(GO:0042742)	FILp
AT1G02205	*Fatty acid hydroxylase superfamily*	defense response to fungus(GO:0050832)	KAN1
AT1G73620	*Pathogenesis-related thaumatin superfamily protein*	response to biotic stimulus(GO:0043207)	KAN1
AT1G75830	*Defensin-like protein 13*	defense response(GO:0006952)	KAN1
AT3G01500	*Beta carbonic anhydrase 1, chloroplastic*	defense response to bacterium(GO:0042742)	KAN1
AT3G13790	*insoluble isoenzyme CWINV1*	defense response to fungus(GO:0050832)	KAN1
AT3G26470	*Powdery mildew resistance protein containing protein*	defense response to fungus(GO:0050832)	KAN1
AT3G45140	*Lipoxygenase 2, chloroplastic*	response to jasmonic acid(GO:0009753)	KAN1
AT4G02410	*L-type lectin-domain containing receptor kinase IV.3*	defense response to bacterium(GO:0042742)	KAN1
AT4G23670	*AT4G23670 protein*	defense response to bacterium(GO:0042742)	KAN1
AT5G14740	*Beta carbonic anhydrase 2, chloroplastic*	defense response to bacterium(GO:0042742)	KAN1
AT1G65390	*Protein PHLOEM PROTEIN 2-LIKE A5*	defense response(GO:0006952)	WUS
AT1G73805	*Protein SAR DEFICIENT 1*	response to biotic stimulus(GO:0002833)	WUS
AT3G29160	*Non-specific serine/threonine protein kinase*	immune effector process(GO:0002697)	WUS
AT5G39580	*Peroxidase 62*	defense response to fungus(GO:0050832)	WUS
AT5G52450	*Protein DETOXIFICATION*	response to biotic stimulus(GO:0043207)	WUS

## Supplementary Materials

Supplementary materials can be found at https://www.mdpi.com/1422-0067/21/16/5745/s1.
