# Supplementary material for: Integrated Framework of the Immune-Defense Transcriptional Signatures in the Arabidopsis Shoot Apical Meristem
Source: Int J Mol Sci. 2020 Aug 11;21(16):5745. doi: 10.3390/ijms21165745 (PMC7460820; doi:10.3390/ijms21165745)
Supplement: Supplementary file 1 [file ijms-21-05745-s001.zip › Supplementary material/Supplementary Table 2.docx]

| **TAIR ID** | **GENE DESCRIPTION** | **GO TERM** | **CELL POPULATION** |
| --- | --- | --- | --- |
| AT1G01470 | LSR3 | defense response to fungus(GO:0050832) | ATHB8 |
| AT1G02660 | Phospholipase A1 PLIP2, chloroplastic | defense response to insect(GO:0002213) | ATHB8 |
| AT1G02860 | E3 ubiquitin-protein ligase BAH1 | salicylic acid biosynthetic process(GO:0009697) | ATHB8 |
| AT1G02930 | Glutathione S-transferase F6 | defense response to bacterium(GO:0042742) | ATHB8 |
| AT1G03830 | Guanylate-binding family protein | immune system process(GO:0002376) | ATHB8 |
| AT1G05010 | 1-aminocyclopropane-1-carboxylate oxidase 4 | response to external biotic stimulus(GO:0043207) | ATHB8 |
| AT1G05690 | BTB/POZ and TAZ domain-containing protein 3 | response to salicylic acid(GO:0009751) | ATHB8 |
| AT1G05800 | Galactolipase DONGLE, chloroplastic | jasmonic acid biosynthetic process(GO:0009695) | ATHB8 |
| AT1G06180 | Transcription factor MYB13 | response to jasmonic acid(GO:0009753) | ATHB8 |
| AT1G07000 | Exocyst subunit Exo70 family protein | response to external biotic stimulus(GO:0043207) | ATHB8 |
| AT1G10170 | NF-X-like 1 | defense response to bacterium(GO:0042742) | ATHB8 |
| AT1G14410 | Single-stranded DNA-binding protein WHY1, chloroplastic | defense response(GO:0006952) | ATHB8 |
| AT1G15010 | Mediator of RNA polymerase II transcription subunit | defense response to fungus(GO:0050832) | ATHB8 |
| AT1G15520 | ABC transporter G family member 40 | response to jasmonic acid(GO:0009753) | ATHB8 |
| AT1G16030 | Hsp70b | response to external biotic stimulus(GO:0043207) | ATHB8 |
| AT1G16310 | Metal tolerance protein 10 | regulation of jasmonic acid mediated signaling pathway(GO:2000022) | ATHB8 |
| AT1G17380 | Protein TIFY 11A | regulation of jasmonic acid mediated signaling pathway(GO:2000022) | ATHB8 |
| AT1G17420 | Lipoxygenase 3, chloroplastic | jasmonic acid biosynthetic process(GO:0009695) | ATHB8 |
| AT1G17860 | Kunitz trypsin inhibitor 2 | response to external biotic stimulus(GO:0043207) | ATHB8 |
| AT1G19180 | TIFY10A | regulation of jasmonic acid mediated signaling pathway(GO:2000022) | ATHB8 |
| AT1G20440 | RD17 | defense response to fungus(GO:0050832) | ATHB8 |
| AT1G20823 | RING-H2 finger protein ATL80 | defense response(GO:0006952) | ATHB8 |
| AT1G20840 | TMT1 | response to external biotic stimulus(GO:0043207) | ATHB8 |
| AT1G20850 | Cysteine protease XCP2 | defense response to bacterium(GO:0042742) | ATHB8 |
| AT1G21250 | Wall-associated receptor kinase 1 | response to salicylic acid(GO:0009751) | ATHB8 |
| AT1G24150 | formin homologue 4 | defense response by cell wall thickening(GO:0052482) | ATHB8 |
| AT1G27950 | Non-specific lipid transfer protein GPI-anchored 1 | defense response to fungus(GO:0050832) | ATHB8 |
| AT1G30135 | Protein TIFY 5A | regulation of jasmonic acid mediated signaling pathway(GO:2000022) | ATHB8 |
| AT1G31290 | Protein argonaute 3 | defense response to other organism(GO:0098542) | ATHB8 |
| AT1G33560 | Disease resistance protein ADR1 | defense response to bacterium(GO:0042742) | ATHB8 |
| AT1G33990 | Putative methylesterase 14, chloroplastic | jasmonic acid metabolic process(GO:0009694) | ATHB8 |
| AT1G43160 | Ethylene-responsive transcription factor RAP2-6 | response to salicylic acid(GO:0009751) | ATHB8 |
| AT1G44350 | IAA-amino acid hydrolase | jasmonic acid metabolic process(GO:0009694) | ATHB8 |
| AT1G45145 | Thioredoxin H5 | defense response to fungus(GO:0050832) | ATHB8 |
| AT1G48000 | MYB transcription factor | response to salicylic acid(GO:0009751) | ATHB8 |
| AT1G49050 | Aspartyl protease APCB1 | defense response to fungus(GO:0050832) | ATHB8 |
| AT1G49430 | Long chain acyl-CoA synthetase 2 | defense response to fungus(GO:0050832) | ATHB8 |
| AT1G51680 | 4-coumarate--CoA ligase 1 | response to external biotic stimulus(GO:0043207) | ATHB8 |
| AT1G52030 | Myrosinase-binding protein 2 | defense response(GO:0006952) | ATHB8 |
| AT1G52400 | Beta-D-glucopyranosyl abscisate beta-glucosidase | defense response to fungus(GO:0050832) | ATHB8 |
| AT1G52410 | TSK-associating protein 1 | defense response to fungus(GO:0050832) | ATHB8 |
| AT1G55020 | Linoleate 9S-lipoxygenase 1 | jasmonic acid biosynthetic process(GO:0009695) | ATHB8 |
| AT1G55210 | Dirigent protein 20 | defense response(GO:0006952) | ATHB8 |
| AT1G58170 | Dirigent protein 19 | defense response(GO:0006952) | ATHB8 |
| AT1G58340 | Protein DETOXIFICATION 48 | response to external biotic stimulus(GO:0043207) | ATHB8 |
| AT1G58410 | Probable disease resistance protein RXW24L | defense response(GO:0006952) | ATHB8 |
| AT1G62380 | 1-aminocyclopropane-1-carboxylate oxidase 2 | defense response(GO:0006952) | ATHB8 |
| AT1G64060 | Respiratory burst oxidase homolog protein F | defense response by callose deposition(GO:0052542) | ATHB8 |
| AT1G64780 | Ammonium transporter 1 member 2 | response to external biotic stimulus(GO:0043207) | ATHB8 |
| AT1G66980 | suppressor of npr1-1 constitutive 4 | defense response(GO:0006952) | ATHB8 |
| AT1G69850 | Protein NRT1/ PTR FAMILY 4.6 | response to external biotic stimulus(GO:0043207) | ATHB8 |
| AT1G70560 | L-tryptophan--pyruvate aminotransferase 1 | defense response to bacterium(GO:0042742) | ATHB8 |
| AT1G70700 | TIFY7 | regulation of jasmonic acid mediated signaling pathway(GO:2000022) | ATHB8 |
| AT1G71930 | NAC domain-containing protein 30 | defense response to fungus(GO:0050832) | ATHB8 |
| AT1G72450 | TIFY11B | regulation of jasmonic acid mediated signaling pathway(GO:2000022) | ATHB8 |
| AT1G72920 | Similar to part of disease resistance protein | defense response(GO:0006952) | ATHB8 |
| AT1G72940 | At1g72940/F3N23_14 | defense response(GO:0006952) | ATHB8 |
| AT1G73080 | Leucine-rich repeat receptor-like protein kinase PEPR1 | response to jasmonic acid(GO:0009753) | ATHB8 |
| AT1G73500 | Mitogen-activated protein kinase kinase 9 | response to external biotic stimulus(GO:0043207) | ATHB8 |
| AT1G74360 | Probable LRR receptor-like serine/threonine-protein kinase | response to external biotic stimulus(GO:0043207) | ATHB8 |
| AT1G74930 | Ethylene-responsive transcription factor ERF018 | defense response to insect(GO:0002213) | ATHB8 |
| AT1G74950 | TIFY10B | regulation of jasmonic acid mediated signaling pathway(GO:2000022) | ATHB8 |
| AT1G76680 | 12-oxophytodienoate reductase 1 | response to salicylic acid(GO:0009751) | ATHB8 |
| AT1G77630 | LysM domain-containing GPI-anchored protein 3 | immune system process(GO:0002376) | ATHB8 |
| AT1G77920 | Transcription factor TGA7 | defense response to bacterium(GO:0042742) | ATHB8 |
| AT2G05940 | Serine/threonine-protein kinase RIPK | defense response to bacterium(GO:0042742) | ATHB8 |
| AT2G06050 | 12-oxophytodienoate reductase 3 | jasmonic acid biosynthetic process(GO:0009695) | ATHB8 |
| AT2G15480 | Glycosyltransferase | response to external biotic stimulus(GO:0043207) | ATHB8 |
| AT2G15890 | CCG-binding protein 1 | defense response to fungus(GO:0050832) | ATHB8 |
| AT2G16720 | Transcription factor MYB7 | response to salicylic acid(GO:0009751) | ATHB8 |
| AT2G18280 | Tubby-like F-box protein | response to external biotic stimulus(GO:0043207) | ATHB8 |
| AT2G18730 | Diacylglycerol kinase 3 | defense response(GO:0006952) | ATHB8 |
| AT2G20370 | Xyloglucan galactosyltransferase MUR3 | response to salicylic acid(GO:0009751) | ATHB8 |
| AT2G21050 | Auxin transporter-like protein 2 | response to external biotic stimulus(GO:0043207) | ATHB8 |
| AT2G22230 | At2g22230/T26C19.11 | defense response to fungus(GO:0050832) | ATHB8 |
| AT2G26330 | LRR receptor-like serine/threonine-protein kinase ERECTA | defense response to bacterium(GO:0042742) | ATHB8 |
| AT2G26690 | Protein NRT1/ PTR FAMILY 6.2 | response to jasmonic acid(GO:0009753) | ATHB8 |
| AT2G27040 | Protein argonaute 4 | defense response to bacterium(GO:0042742) | ATHB8 |
| AT2G29420 | Glutathione S-transferase U7 | response to salicylic acid(GO:0009751) | ATHB8 |
| AT2G32800 | Receptor like protein kinase S.2 | response to jasmonic acid(GO:0009753) | ATHB8 |
| AT2G33380 | Probable peroxygenase 3 | defense response to fungus(GO:0050832) | ATHB8 |
| AT2G34930 | Disease resistance family protein / LRR family protein | defense response to fungus(GO:0050832) | ATHB8 |
| AT2G35940 | BEL1-like homeodomain protein 1 | response to external biotic stimulus(GO:0043207) | ATHB8 |
| AT2G35980 | NDR1/HIN1-like protein 10 | defense response to other organism(GO:0098542) | ATHB8 |
| AT2G38120 | Auxin transporter protein 1 | response to external biotic stimulus(GO:0043207) | ATHB8 |
| AT2G38240 | Probable 2-oxoglutarate-dependent dioxygenase ANS | regulation of jasmonic acid mediated signaling pathway(GO:2000022) | ATHB8 |
| AT2G39200 | MLO-like protein | defense response to fungus(GO:0050832) | ATHB8 |
| AT2G40460 | Protein NRT1/ PTR FAMILY 5.1 | response to external biotic stimulus(GO:0043207) | ATHB8 |
| AT2G42530 | Protein COLD-REGULATED 15B, chloroplastic | defense response to fungus(GO:0050832) | ATHB8 |
| AT2G43520 | TI2 | defense response(GO:0006952) | ATHB8 |
| AT2G44490 | Beta-glucosidase 26, peroxisomal | defense response by callose deposition in cell wall(GO:0052544) | ATHB8 |
| AT2G46370 | Auxin-responsive GH3 family protein | defense response to bacterium(GO:0042742) | ATHB8 |
| AT3G05180 | GDSL esterase/lipase At3g05180 | defense response to other organism(GO:0098542) | ATHB8 |
| AT3G05200 | E3 ubiquitin-protein ligase ATL6 | defense response to bacterium(GO:0042742) | ATHB8 |
| AT3G05650 | Receptor-like protein 32 | defense response(GO:0006952) | ATHB8 |
| AT3G06490 | Transcription factor MYB108 | response to jasmonic acid(GO:0009753) | ATHB8 |
| AT3G09440 | Heat shock protein 70 (Hsp 70) family protein | response to external biotic stimulus(GO:0043207) | ATHB8 |
| AT3G10525 | SMR1 | defense response to bacterium(GO:0042742) | ATHB8 |
| AT3G10985 | Senescence associated gene 20 | response to external biotic stimulus(GO:0043207) | ATHB8 |
| AT3G11840 | E3 ubiquitin-protein ligase PUB24 | immune effector process(GO:0002252) | ATHB8 |
| AT3G12580 | Probable mediator of RNA polymerase II transcription subunit 37c | response to external biotic stimulus(GO:0043207) | ATHB8 |
| AT3G13100 | ABC transporter C family member 7 | response to external biotic stimulus(GO:0043207) | ATHB8 |
| AT3G13650 | Dirigent protein 7 | defense response(GO:0006952) | ATHB8 |
| AT3G13660 | Dirigent protein 22 | defense response(GO:0006952) | ATHB8 |
| AT3G15020 | Malate dehydrogenase 2, mitochondrial | defense response to bacterium(GO:0042742) | ATHB8 |
| AT3G15210 | Ethylene-responsive transcription factor 4 | defense response to bacterium(GO:0042742) | ATHB8 |
| AT3G15500 | NAC3 | response to jasmonic acid(GO:0009753) | ATHB8 |
| AT3G16470 | Jacalin-related lectin 35 | response to jasmonic acid(GO:0009753) | ATHB8 |
| AT3G16770 | Ethylene-responsive transcription factor RAP2-3 | response to jasmonic acid(GO:0009753) | ATHB8 |
| AT3G17860 | Protein TIFY 6B | regulation of jasmonic acid mediated signaling pathway(GO:2000022) | ATHB8 |
| AT3G20250 | Pumilio 5 | defense response to other organism(GO:0098542) | ATHB8 |
| AT3G21240 | Cinnamyl alcohol dehydrogenase | response to external biotic stimulus(GO:0043207) | ATHB8 |
| AT3G23170 | At3g23170 | defense response to other organism(GO:0098542) | ATHB8 |
| AT3G23250 | Transcription factor MYB15 | response to jasmonic acid(GO:0009753) | ATHB8 |
| AT3G25250 | Serine/threonine-protein kinase OXI1 | defense response(GO:0006952) | ATHB8 |
| AT3G25760 | At3g25760 | jasmonic acid biosynthetic process(GO:0009695) | ATHB8 |
| AT3G25780 | Allene oxide cyclase 3, chloroplastic | jasmonic acid biosynthetic process(GO:0009695) | ATHB8 |
| AT3G27400 | Pectate lyase | response to external biotic stimulus(GO:0043207) | ATHB8 |
| AT3G28740 | Cytochrome P450 81D11 | defense response to insect(GO:0002213) | ATHB8 |
| AT3G29770 | Putative methylesterase 11, chloroplastic | jasmonic acid metabolic process(GO:0009694) | ATHB8 |
| AT3G47600 | Transcription factor MYB94 | response to salicylic acid(GO:0009751) | ATHB8 |
| AT3G48360 | BTB/POZ and TAZ domain-containing protein 2 | response to salicylic acid(GO:0009751) | ATHB8 |
| AT3G48520 | CYP94B3 | defense response to insect(GO:0002213) | ATHB8 |
| AT3G50950 | Disease resistance RPP13-like protein 4 | defense response to bacterium(GO:0042742) | ATHB8 |
| AT3G50970 | Dehydrin Xero 2 | defense response to fungus(GO:0050832) | ATHB8 |
| AT3G51260 | Proteasome subunit alpha type | defense response to bacterium(GO:0042742) | ATHB8 |
| AT3G51860 | Vacuolar cation/proton exchanger | response to external biotic stimulus(GO:0043207) | ATHB8 |
| AT3G51870 | Probable envelope ADP,ATP carrier protein, chloroplastic | response to external biotic stimulus(GO:0043207) | ATHB8 |
| AT3G52430 | Lipase-like PAD4 | defense response to insect(GO:0002213) | ATHB8 |
| AT3G52450 | RING-type E3 ubiquitin transferase | immune effector process(GO:0002252) | ATHB8 |
| AT3G52960 | Peroxiredoxin-2E, chloroplastic | defense response to bacterium(GO:0042742) | ATHB8 |
| AT3G53810 | L-type lectin-domain containing receptor kinase IV.2 | defense response to bacterium(GO:0042742) | ATHB8 |
| AT3G53980 | At3g53980 | defense response to other organism(GO:0098542) | ATHB8 |
| AT3G54560 | Histone H2A | defense response to bacterium(GO:0042742) | ATHB8 |
| AT3G54950 | Patatin-like protein 7 | response to external biotic stimulus(GO:0043207) | ATHB8 |
| AT3G55970 | JRG21 | regulation of jasmonic acid mediated signaling pathway(GO:2000022) | ATHB8 |
| AT3G59750 | Putative L-type lectin-domain containing receptor kinase V.8 | defense response to bacterium(GO:0042742) | ATHB8 |
| AT4G02380 | senescence-associated gene 21 | response to external biotic stimulus(GO:0043207) | ATHB8 |
| AT4G04960 | L-type lectin-domain containing receptor kinase VII.1 | defense response to bacterium(GO:0042742) | ATHB8 |
| AT4G05100 | Transcription factor MYB74 | response to jasmonic acid(GO:0009753) | ATHB8 |
| AT4G14940 | Primary amine oxidase 1 | response to jasmonic acid(GO:0009753) | ATHB8 |
| AT4G18780 | Cellulose synthase (Fragment) | defense response to bacterium(GO:0042742) | ATHB8 |
| AT4G19230 | Cytochrome P450, family 707, subfamily A, polypeptide 1 | defense response to fungus(GO:0050832) | ATHB8 |
| AT4G20830 | Berberine bridge enzyme-like 19 | defense response to fungus(GO:0050832) | ATHB8 |
| AT4G23180 | Cysteine-rich receptor-like protein kinase 10 | defense response to bacterium(GO:0042742) | ATHB8 |
| AT4G23690 | Dirigent protein 6 | defense response(GO:0006952) | ATHB8 |
| AT4G23810 | Probable WRKY transcription factor 53 | regulation of jasmonic acid mediated signaling pathway(GO:2000022) | ATHB8 |
| AT4G31800 | WRKY like transcription factor | response to salicylic acid(GO:0009751) | ATHB8 |
| AT4G32940 | GAMMAVPE | response to jasmonic acid(GO:0009753) | ATHB8 |
| AT4G33300 | Probable disease resistance protein At4g33300 | defense response to bacterium(GO:0042742) | ATHB8 |
| AT4G34135 | UDP-glucosyl transferase 73B2 | response to external biotic stimulus(GO:0043207) | ATHB8 |
| AT4G36010 | Pathogenesis-related thaumatin superfamily protein | response to external biotic stimulus(GO:0043207) | ATHB8 |
| AT4G37760 | Squalene epoxidase 3 | response to jasmonic acid(GO:0009753) | ATHB8 |
| AT4G37870 | Phosphoenolpyruvate carboxykinase (ATP)-like protein | defense response to fungus(GO:0050832) | ATHB8 |
| AT5G01600 | Ferritin-1, chloroplastic | response to external biotic stimulus(GO:0043207) | ATHB8 |
| AT5G02490 | Probable mediator of RNA polymerase II transcription subunit 37c | response to external biotic stimulus(GO:0043207) | ATHB8 |
| AT5G03210 | Arabidopsis thaliana genomic DNA, chromosome 5, P1 clone:MOK16 | defense response to other organism(GO:0098542) | ATHB8 |
| AT5G03760 | Glucomannan 4-beta-mannosyltransferase 9 | response to external biotic stimulus(GO:0043207) | ATHB8 |
| AT5G03780 | TRF-like 10 (Fragment) | response to salicylic acid(GO:0009751) | ATHB8 |
| AT5G04760 | Duplicated homeodomain-like superfamily protein | response to salicylic acid(GO:0009751) | ATHB8 |
| AT5G06290 | 2-Cys peroxiredoxin BAS1-like, chloroplastic | defense response to bacterium(GO:0042742) | ATHB8 |
| AT5G06860 | PGIP1 | defense response(GO:0006952) | ATHB8 |
| AT5G06870 | PGIP2 | defense response(GO:0006952) | ATHB8 |
| AT5G07010 | Sulfotransferase | jasmonic acid metabolic process(GO:0009694) | ATHB8 |
| AT5G07920 | Diacylglycerol kinase 1 | defense response(GO:0006952) | ATHB8 |
| AT5G08370 | Alpha-galactosidase 2 | response to external biotic stimulus(GO:0043207) | ATHB8 |
| AT5G08640 | Flavonol synthase/flavanone 3-hydroxylase | response to external biotic stimulus(GO:0043207) | ATHB8 |
| AT5G10520 | RBK1 | defense response to other organism(GO:0098542) | ATHB8 |
| AT5G13330 | Rap2.6L | response to jasmonic acid(GO:0009753) | ATHB8 |
| AT5G13930 | Chalcone synthase family protein | response to jasmonic acid(GO:0009753) | ATHB8 |
| AT5G14180 | Triacylglycerol lipase 2 | defense response to insect(GO:0002213) | ATHB8 |
| AT5G15090 | Mitochondrial outer membrane protein porin 3 | defense response to bacterium(GO:0042742) | ATHB8 |
| AT5G16000 | Protein NSP-INTERACTING KINASE 1 | defense response to other organism(GO:0098542) | ATHB8 |
| AT5G17490 | DELLA protein RGL3 | response to jasmonic acid(GO:0009753) | ATHB8 |
| AT5G18860 | AT5g18860/F17K4_110 | response to jasmonic acid(GO:0009753) | ATHB8 |
| AT5G24090 | Acidic endochitinase | response to external biotic stimulus(GO:0043207) | ATHB8 |
| AT5G27420 | E3 ubiquitin-protein ligase ATL31 | defense response to bacterium(GO:0042742) | ATHB8 |
| AT5G37260 | Protein REVEILLE 2 | response to salicylic acid(GO:0009751) | ATHB8 |
| AT5G40770 | Prohibitin-3, mitochondrial | salicylic acid biosynthetic process(GO:0009697) | ATHB8 |
| AT5G40910 | Disease resistance protein (TIR-NBS-LRR class) family | defense response(GO:0006952) | ATHB8 |
| AT5G42650 | Allene oxide synthase, chloroplastic | jasmonic acid biosynthetic process(GO:0009695) | ATHB8 |
| AT5G43810 | Protein argonaute 10 | defense response to other organism(GO:0098542) | ATHB8 |
| AT5G44030 | cellulose synthase A4 | defense response to fungus(GO:0050832) | ATHB8 |
| AT5G44510 | Disease resistance protein TAO1 | defense response to bacterium(GO:0042742) | ATHB8 |
| AT5G45800 | MEE62 | response to external biotic stimulus(GO:0043207) | ATHB8 |
| AT5G46330 | Leucine-rich repeat receptor-like protein kinase (Fragment) | defense response by callose deposition in cell wall(GO:0052544) | ATHB8 |
| AT5G47250 | Probable disease resistance protein At5g47250 | defense response(GO:0006952) | ATHB8 |
| AT5G52310 | Low-temperature-induced 78 kDa protein | response to external biotic stimulus(GO:0043207) | ATHB8 |
| AT5G53550 | YSL3 | response to external biotic stimulus(GO:0043207) | ATHB8 |
| AT5G58120 | Disease resistance protein (TIR-NBS-LRR class) family | defense response(GO:0006952) | ATHB8 |
| AT5G62380 | NAC domain-containing protein 101 | response to external biotic stimulus(GO:0043207) | ATHB8 |
| AT5G62470 | Transcription factor MYB96 | response to salicylic acid(GO:0009751) | ATHB8 |
| AT5G63450 | cytochrome P450, family 94, subfamily B, polypeptide 1 | jasmonic acid metabolic process(GO:0009694) | ATHB8 |
| AT5G63770 | Diacylglycerol kinase 2 | defense response(GO:0006952) | ATHB8 |
| AT5G63970 | E3 ubiquitin-protein ligase RGLG3 | defense response to bacterium(GO:0042742) | ATHB8 |
| AT5G65040 | Protein INCREASED RESISTANCE TO MYZUS PERSICAE 1 | defense response to insect(GO:0002213) | ATHB8 |
| AT5G65210 | Transcription factor TGA1 | defense response to bacterium(GO:0042742) | ATHB8 |
| AT5G65530 | Protein kinase superfamily protein | defense response to fungus(GO:0050832) | ATHB8 |
| AT5G67300 | MYBR1 | regulation of jasmonic acid mediated signaling pathway(GO:2000022) | ATHB8 |
| AT1G01470 | LSR3 | defense response to fungus(GO:0050832) | S17 |
| AT1G02305 | Cathepsin B-like protease 2 | defense response(GO:0006952) | S17 |
| AT1G02800 | Endoglucanase 1 | response to external biotic stimulus(GO:0043207) | S17 |
| AT1G02860 | E3 ubiquitin-protein ligase BAH1 | salicylic acid biosynthetic process(GO:0009697) | S17 |
| AT1G03830 | Guanylate-binding family protein | immune system process(GO:0002376) | S17 |
| AT1G04400 | Cryptochrome-2 | response to external biotic stimulus(GO:0043207) | S17 |
| AT1G04510 | Pre-mRNA-processing factor 19 homolog 1 | defense response to bacterium(GO:0042742) | S17 |
| AT1G05560 | UDP-glucosyltransferase 75B1 | response to salicylic acid(GO:0009751) | S17 |
| AT1G07640 | Dof-type zinc finger DNA-binding family protein | response to jasmonic acid(GO:0009753) | S17 |
| AT1G11000 | MLO-like protein 4 | defense response(GO:0006952) | S17 |
| AT1G11580 | methylesterase PCR A | response to external biotic stimulus(GO:0043207) | S17 |
| AT1G12200 | Flavin-containing monooxygenase | defense response to fungus(GO:0050832) | S17 |
| AT1G12240 | Acid beta-fructofuranosidase 4, vacuolar | response to external biotic stimulus(GO:0043207) | S17 |
| AT1G13280 | Allene oxide cyclase 4, chloroplastic | jasmonic acid biosynthetic process(GO:0009695) | S17 |
| AT1G14780 | MACPF domain-containing protein At1g14780 | defense response(GO:0006952) | S17 |
| AT1G14790 | RNA-dependent RNA polymerase | response to salicylic acid(GO:0009751) | S17 |
| AT1G15010 | Mediator of RNA polymerase II transcription subunit | defense response to fungus(GO:0050832) | S17 |
| AT1G15380 | GLYI4 | regulation of jasmonic acid mediated signaling pathway(GO:2000022) | S17 |
| AT1G16070 | TLP8 | response to external biotic stimulus(GO:0043207) | S17 |
| AT1G16310 | Metal tolerance protein 10 | regulation of jasmonic acid mediated signaling pathway(GO:2000022) | S17 |
| AT1G17380 | Protein TIFY 11A | regulation of jasmonic acid mediated signaling pathway(GO:2000022) | S17 |
| AT1G17420 | Lipoxygenase 3, chloroplastic | jasmonic acid biosynthetic process(GO:0009695) | S17 |
| AT1G17750 | Leucine-rich repeat receptor-like protein kinase PEPR2 | defense response to bacterium(GO:0042742) | S17 |
| AT1G18570 | Transcription factor MYB51 | defense response by callose deposition in cell wall(GO:0052544) | S17 |
| AT1G18710 | MYB transcription factor | response to jasmonic acid(GO:0009753) | S17 |
| AT1G20030 | Pathogenesis-related thaumatin superfamily protein | response to external biotic stimulus(GO:0043207) | S17 |
| AT1G20823 | RING-H2 finger protein ATL80 | defense response(GO:0006952) | S17 |
| AT1G22070 | At1g22070 | defense response to bacterium(GO:0042742) | S17 |
| AT1G24100 | Glycosyltransferase | defense response by callose deposition in cell wall(GO:0052544) | S17 |
| AT1G26190 | Inorganic pyrophosphatase TTM2 | regulation of jasmonic acid mediated signaling pathway(GO:2000022) | S17 |
| AT1G27130 | Glutathione S-transferase U13 | defense response to fungus(GO:0050832) | S17 |
| AT1G27950 | Non-specific lipid transfer protein GPI-anchored 1 | defense response to fungus(GO:0050832) | S17 |
| AT1G30135 | Protein TIFY 5A | regulation of jasmonic acid mediated signaling pathway(GO:2000022) | S17 |
| AT1G31290 | Protein argonaute 3 | defense response to other organism(GO:0098542) | S17 |
| AT1G33590 | Leucine-rich repeat (LRR) family protein | defense response(GO:0006952) | S17 |
| AT1G33990 | Putative methylesterase 14, chloroplastic | jasmonic acid metabolic process(GO:0009694) | S17 |
| AT1G44350 | IAA-amino acid hydrolase | jasmonic acid metabolic process(GO:0009694) | S17 |
| AT1G45145 | Thioredoxin H5 | defense response to fungus(GO:0050832) | S17 |
| AT1G47128 | RD21A | defense response to fungus(GO:0050832) | S17 |
| AT1G49010 | At1g49010 | response to jasmonic acid(GO:0009753) | S17 |
| AT1G49430 | Long chain acyl-CoA synthetase 2 | defense response to fungus(GO:0050832) | S17 |
| AT1G51680 | 4-coumarate--CoA ligase 1 | response to external biotic stimulus(GO:0043207) | S17 |
| AT1G52030 | Myrosinase-binding protein 2 | defense response(GO:0006952) | S17 |
| AT1G52400 | Beta-D-glucopyranosyl abscisate beta-glucosidase | defense response to fungus(GO:0050832) | S17 |
| AT1G52410 | TSK-associating protein 1 | defense response to fungus(GO:0050832) | S17 |
| AT1G54040 | Epithiospecifier protein | response to jasmonic acid(GO:0009753) | S17 |
| AT1G55210 | Dirigent protein 20 | defense response(GO:0006952) | S17 |
| AT1G58170 | Dirigent protein 19 | defense response(GO:0006952) | S17 |
| AT1G58340 | Protein DETOXIFICATION 48 | response to external biotic stimulus(GO:0043207) | S17 |
| AT1G58360 | NAT2 | response to external biotic stimulus(GO:0043207) | S17 |
| AT1G58410 | Probable disease resistance protein RXW24L | defense response(GO:0006952) | S17 |
| AT1G59620 | Disease resistance protein (CC-NBS-LRR class) family | defense response(GO:0006952) | S17 |
| AT1G59740 | Protein NRT1/ PTR FAMILY 4.3 | response to external biotic stimulus(GO:0043207) | S17 |
| AT1G60960 | IRT3 | response to external biotic stimulus(GO:0043207) | S17 |
| AT1G62380 | 1-aminocyclopropane-1-carboxylate oxidase 2 | defense response(GO:0006952) | S17 |
| AT1G62660 | Acid beta-fructofuranosidase 3, vacuolar | response to external biotic stimulus(GO:0043207) | S17 |
| AT1G64060 | Respiratory burst oxidase homolog protein F | defense response by callose deposition(GO:0052542) | S17 |
| AT1G64280 | Regulatory protein NPR1 | regulation of jasmonic acid mediated signaling pathway(GO:2000022) | S17 |
| AT1G64780 | Ammonium transporter 1 member 2 | response to external biotic stimulus(GO:0043207) | S17 |
| AT1G66350 | DELLA protein RGL1 | response to salicylic acid(GO:0009751) | S17 |
| AT1G66980 | suppressor of npr1-1 constitutive 4 | defense response(GO:0006952) | S17 |
| AT1G67560 | Lipoxygenase | jasmonic acid biosynthetic process(GO:0009695) | S17 |
| AT1G67710 | Two-component response regulator | regulation of jasmonic acid mediated signaling pathway(GO:2000022) | S17 |
| AT1G67865 | At1g67862/At1g67862 | defense response to fungus(GO:0050832) | S17 |
| AT1G69170 | Squamosa promoter-binding-like protein 6 | defense response to bacterium(GO:0042742) | S17 |
| AT1G69850 | Protein NRT1/ PTR FAMILY 4.6 | response to external biotic stimulus(GO:0043207) | S17 |
| AT1G70000 | At1g70000 | response to salicylic acid(GO:0009751) | S17 |
| AT1G70130 | Putative L-type lectin-domain containing receptor kinase V.2 | defense response to bacterium(GO:0042742) | S17 |
| AT1G70700 | TIFY7 | regulation of jasmonic acid mediated signaling pathway(GO:2000022) | S17 |
| AT1G72450 | TIFY11B | regulation of jasmonic acid mediated signaling pathway(GO:2000022) | S17 |
| AT1G72520 | Lipoxygenase 4, chloroplastic | jasmonic acid biosynthetic process(GO:0009695) | S17 |
| AT1G74020 | Protein STRICTOSIDINE SYNTHASE-LIKE 12 | response to jasmonic acid(GO:0009753) | S17 |
| AT1G74100 | Sulfotransferase | response to jasmonic acid(GO:0009753) | S17 |
| AT1G74930 | Ethylene-responsive transcription factor ERF018 | defense response to insect(GO:0002213) | S17 |
| AT1G77470 | Replication factor C subunit 5 | defense response(GO:0006952) | S17 |
| AT1G77630 | LysM domain-containing GPI-anchored protein 3 | immune system process(GO:0002376) | S17 |
| AT1G77920 | Transcription factor TGA7 | defense response to bacterium(GO:0042742) | S17 |
| AT2G02130 | Defensin-like protein 1 | defense response(GO:0006952) | S17 |
| AT2G05940 | Serine/threonine-protein kinase RIPK | defense response to bacterium(GO:0042742) | S17 |
| AT2G06050 | 12-oxophytodienoate reductase 3 | jasmonic acid biosynthetic process(GO:0009695) | S17 |
| AT2G16720 | Transcription factor MYB7 | response to salicylic acid(GO:0009751) | S17 |
| AT2G18280 | Tubby-like F-box protein | response to external biotic stimulus(GO:0043207) | S17 |
| AT2G22230 | At2g22230/T26C19.11 | defense response to fungus(GO:0050832) | S17 |
| AT2G22330 | cytochrome P450, family 79, subfamily B, polypeptide 3 | defense response by callose deposition in cell wall(GO:0052544) | S17 |
| AT2G22370 | MED18 | regulation of jasmonic acid mediated signaling pathway(GO:2000022) | S17 |
| AT2G22840 | Growth-regulating factor 1 | response to external biotic stimulus(GO:0043207) | S17 |
| AT2G23610 | MES3 | salicylic acid metabolic process(GO:0009696) | S17 |
| AT2G26690 | Protein NRT1/ PTR FAMILY 6.2 | response to jasmonic acid(GO:0009753) | S17 |
| AT2G27040 | Protein argonaute 4 | defense response to bacterium(GO:0042742) | S17 |
| AT2G28590 | Probable serine/threonine-protein kinase PBL6 | defense response(GO:0006952) | S17 |
| AT2G28900 | Outer envelope pore protein 16-1, chloroplastic | response to jasmonic acid(GO:0009753) | S17 |
| AT2G29630 | Phosphomethylpyrimidine synthase, chloroplastic | response to external biotic stimulus(GO:0043207) | S17 |
| AT2G30080 | ZIP6 | response to external biotic stimulus(GO:0043207) | S17 |
| AT2G32140 | Transmembrane receptor | defense response(GO:0006952) | S17 |
| AT2G32800 | Receptor like protein kinase S.2 | response to jasmonic acid(GO:0009753) | S17 |
| AT2G32940 | Protein argonaute 6 | defense response to other organism(GO:0098542) | S17 |
| AT2G33380 | Probable peroxygenase 3 | defense response to fungus(GO:0050832) | S17 |
| AT2G34600 | Protein TIFY 5B | regulation of jasmonic acid mediated signaling pathway(GO:2000022) | S17 |
| AT2G34810 | Berberine bridge enzyme-like 16 | response to jasmonic acid(GO:0009753) | S17 |
| AT2G34930 | Disease resistance family protein / LRR family protein | defense response to fungus(GO:0050832) | S17 |
| AT2G35940 | BEL1-like homeodomain protein 1 | response to external biotic stimulus(GO:0043207) | S17 |
| AT2G35960 | NDR1/HIN1-like protein 12 | defense response(GO:0006952) | S17 |
| AT2G35980 | NDR1/HIN1-like protein 10 | defense response to other organism(GO:0098542) | S17 |
| AT2G37040 | Phenylalanine ammonia-lyase 1 | salicylic acid metabolic process(GO:0009696) | S17 |
| AT2G37710 | L-type lectin-domain containing receptor kinase IV.1 | defense response to bacterium(GO:0042742) | S17 |
| AT2G38240 | Probable 2-oxoglutarate-dependent dioxygenase ANS | regulation of jasmonic acid mediated signaling pathway(GO:2000022) | S17 |
| AT2G38870 | Putative protease inhibitor | defense response to fungus(GO:0050832) | S17 |
| AT2G39010 | Probable aquaporin PIP2-6 | response to external biotic stimulus(GO:0043207) | S17 |
| AT2G39660 | BIK1 | defense response to fungus(GO:0050832) | S17 |
| AT2G39770 | VTC1 | defense response to bacterium(GO:0042742) | S17 |
| AT2G40460 | Protein NRT1/ PTR FAMILY 5.1 | response to external biotic stimulus(GO:0043207) | S17 |
| AT2G41560 | Calcium-transporting ATPase 4, plasma membrane-type | defense response to bacterium(GO:0042742) | S17 |
| AT2G42530 | Protein COLD-REGULATED 15B, chloroplastic | defense response to fungus(GO:0050832) | S17 |
| AT2G43530 | Defensin-like protein 194 | defense response to fungus(GO:0050832) | S17 |
| AT2G43550 | Defensin-like protein 197 | defense response to fungus(GO:0050832) | S17 |
| AT2G44490 | Beta-glucosidase 26, peroxisomal | defense response by callose deposition in cell wall(GO:0052544) | S17 |
| AT2G46370 | Auxin-responsive GH3 family protein | defense response to bacterium(GO:0042742) | S17 |
| AT2G46440 | Cyclic nucleotide-gated ion channel 11 | response to external biotic stimulus(GO:0043207) | S17 |
| AT2G47730 | Glutathione S-transferase F8, chloroplastic | defense response to bacterium(GO:0042742) | S17 |
| AT3G01290 | HIR2 | response to external biotic stimulus(GO:0043207) | S17 |
| AT3G03450 | RGL2 | response to salicylic acid(GO:0009751) | S17 |
| AT3G05650 | Receptor-like protein 32 | defense response(GO:0006952) | S17 |
| AT3G05660 | Receptor-like protein 33 | defense response(GO:0006952) | S17 |
| AT3G06490 | Transcription factor MYB108 | response to jasmonic acid(GO:0009753) | S17 |
| AT3G11050 | Ferritin-2, chloroplastic | response to external biotic stimulus(GO:0043207) | S17 |
| AT3G11280 | Duplicated homeodomain-like superfamily protein | response to salicylic acid(GO:0009751) | S17 |
| AT3G11660 | NDR1/HIN1-like protein 1 | defense response to other organism(GO:0098542) | S17 |
| AT3G11840 | E3 ubiquitin-protein ligase PUB24 | immune effector process(GO:0002252) | S17 |
| AT3G12580 | Probable mediator of RNA polymerase II transcription subunit 37c | response to external biotic stimulus(GO:0043207) | S17 |
| AT3G12920 | Probable BOI-related E3 ubiquitin-protein ligase 3 | defense response(GO:0006952) | S17 |
| AT3G13100 | ABC transporter C family member 7 | response to external biotic stimulus(GO:0043207) | S17 |
| AT3G13650 | Dirigent protein 7 | defense response(GO:0006952) | S17 |
| AT3G13660 | Dirigent protein 22 | defense response(GO:0006952) | S17 |
| AT3G13662 | Dirigent protein 8 | defense response(GO:0006952) | S17 |
| AT3G14310 | Pectinesterase/pectinesterase inhibitor 3 | defense response to bacterium(GO:0042742) | S17 |
| AT3G14395 | At3g14395 | response to external biotic stimulus(GO:0043207) | S17 |
| AT3G15020 | Malate dehydrogenase 2, mitochondrial | defense response to bacterium(GO:0042742) | S17 |
| AT3G15500 | NAC3 | response to jasmonic acid(GO:0009753) | S17 |
| AT3G16470 | Jacalin-related lectin 35 | response to jasmonic acid(GO:0009753) | S17 |
| AT3G16770 | Ethylene-responsive transcription factor RAP2-3 | response to jasmonic acid(GO:0009753) | S17 |
| AT3G17860 | Protein TIFY 6B | regulation of jasmonic acid mediated signaling pathway(GO:2000022) | S17 |
| AT3G18690 | Protein MKS1 | immune system process(GO:0002376) | S17 |
| AT3G20250 | Pumilio 5 | defense response to other organism(GO:0098542) | S17 |
| AT3G21240 | Cinnamyl alcohol dehydrogenase | response to external biotic stimulus(GO:0043207) | S17 |
| AT3G23010 | 0 | defense response(GO:0006952) | S17 |
| AT3G23110 | Receptor-like protein 37 | defense response(GO:0006952) | S17 |
| AT3G24800 | E3 ubiquitin-protein ligase PRT1 | defense response to fungus(GO:0050832) | S17 |
| AT3G25560 | NSP-interacting kinase 2 | defense response(GO:0006952) | S17 |
| AT3G25760 | At3g25760 | jasmonic acid biosynthetic process(GO:0009695) | S17 |
| AT3G25780 | Allene oxide cyclase 3, chloroplastic | jasmonic acid biosynthetic process(GO:0009695) | S17 |
| AT3G26520 | Aquaporin TIP1-2 | defense response to bacterium(GO:0042742) | S17 |
| AT3G27810 | Transcription factor MYB21 | response to jasmonic acid(GO:0009753) | S17 |
| AT3G28740 | Cytochrome P450 81D11 | defense response to insect(GO:0002213) | S17 |
| AT3G29770 | Putative methylesterase 11, chloroplastic | jasmonic acid metabolic process(GO:0009694) | S17 |
| AT3G44630 | Disease resistance protein (TIR-NBS-LRR class) family | defense response(GO:0006952) | S17 |
| AT3G45680 | Protein NRT1/ PTR FAMILY 2.3 | response to external biotic stimulus(GO:0043207) | S17 |
| AT3G47600 | Transcription factor MYB94 | response to salicylic acid(GO:0009751) | S17 |
| AT3G48090 | Protein EDS1 | defense response to bacterium(GO:0042742) | S17 |
| AT3G48520 | CYP94B3 | defense response to insect(GO:0002213) | S17 |
| AT3G49110 | Peroxidase 33 | defense response to bacterium(GO:0042742) | S17 |
| AT3G49690 | RAX3 | response to salicylic acid(GO:0009751) | S17 |
| AT3G50410 | OBP1 | response to salicylic acid(GO:0009751) | S17 |
| AT3G50440 | Methylesterase 10 | jasmonic acid metabolic process(GO:0009694) | S17 |
| AT3G50660 | Cytochrome P450 90B1 | response to jasmonic acid(GO:0009753) | S17 |
| AT3G50930 | Protein HYPER-SENSITIVITY-RELATED 4 | response to salicylic acid(GO:0009751) | S17 |
| AT3G52430 | Lipase-like PAD4 | defense response to insect(GO:0002213) | S17 |
| AT3G52960 | Peroxiredoxin-2E, chloroplastic | defense response to bacterium(GO:0042742) | S17 |
| AT3G53260 | Phenylalanine ammonia-lyase 2 | defense response(GO:0006952) | S17 |
| AT3G53980 | At3g53980 | defense response to other organism(GO:0098542) | S17 |
| AT3G54560 | Histone H2A | defense response to bacterium(GO:0042742) | S17 |
| AT3G54950 | Patatin-like protein 7 | response to external biotic stimulus(GO:0043207) | S17 |
| AT3G55970 | JRG21 | regulation of jasmonic acid mediated signaling pathway(GO:2000022) | S17 |
| AT3G56400 | Probable WRKY transcription factor 70 | regulation of jasmonic acid mediated signaling pathway(GO:2000022) | S17 |
| AT3G59750 | Putative L-type lectin-domain containing receptor kinase V.8 | defense response to bacterium(GO:0042742) | S17 |
| AT3G61250 | Transcription factor MYB41 | response to jasmonic acid(GO:0009753) | S17 |
| AT3G61440 | Bifunctional L-3-cyanoalanine synthase/cysteine synthase C1, mitochondrial | immune system process(GO:0002376) | S17 |
| AT3G61890 | HB-12 | response to external biotic stimulus(GO:0043207) | S17 |
| AT4G01050 | thylakoid rhodanese-like | defense response to bacterium(GO:0042742) | S17 |
| AT4G01610 | Cathepsin B-like protease 3 | defense response(GO:0006952) | S17 |
| AT4G01700 | At4g01700 | response to external biotic stimulus(GO:0043207) | S17 |
| AT4G02380 | senescence-associated gene 21 | response to external biotic stimulus(GO:0043207) | S17 |
| AT4G03110 | RNA-binding protein BRN1 | regulation of jasmonic acid mediated signaling pathway(GO:2000022) | S17 |
| AT4G04220 | Receptor-like protein 46 | defense response(GO:0006952) | S17 |
| AT4G09460 | Transcription repressor MYB6 | response to jasmonic acid(GO:0009753) | S17 |
| AT4G13770 | Cytochrome P450 83A1 | response to external biotic stimulus(GO:0043207) | S17 |
| AT4G15210 | Beta-amylase 5 | response to external biotic stimulus(GO:0043207) | S17 |
| AT4G15900 | Protein pleiotropic regulatory locus 1 | defense response to fungus(GO:0050832) | S17 |
| AT4G16950 | Disease resistance protein RPP5 | defense response to fungus(GO:0050832) | S17 |
| AT4G17490 | Ethylene-responsive transcription factor 6 | response to external biotic stimulus(GO:0043207) | S17 |
| AT4G17880 | Transcription factor MYC4 | defense response(GO:0006952) | S17 |
| AT4G18470 | Negative regulator of systemic acquired resistance SNI1 | defense response to other organism(GO:0098542) | S17 |
| AT4G19840 | Protein PHLOEM PROTEIN 2-LIKE A1 | response to external biotic stimulus(GO:0043207) | S17 |
| AT4G25030 | AT4G25030 protein | defense response to bacterium(GO:0042742) | S17 |
| AT4G26090 | Disease resistance protein RPS2 | defense response to bacterium(GO:0042742) | S17 |
| AT4G31500 | Cytochrome P450 83B1 | defense response by callose deposition in cell wall(GO:0052544) | S17 |
| AT4G32940 | GAMMAVPE | response to jasmonic acid(GO:0009753) | S17 |
| AT4G33300 | Probable disease resistance protein At4g33300 | defense response to bacterium(GO:0042742) | S17 |
| AT4G33950 | Serine/threonine-protein kinase SRK2E | defense response to bacterium(GO:0042742) | S17 |
| AT4G34135 | UDP-glucosyl transferase 73B2 | response to external biotic stimulus(GO:0043207) | S17 |
| AT4G35770 | SEN1 | response to jasmonic acid(GO:0009753) | S17 |
| AT4G37760 | Squalene epoxidase 3 | response to jasmonic acid(GO:0009753) | S17 |
| AT4G37930 | Serine hydroxymethyltransferase 1, mitochondrial | immune system process(GO:0002376) | S17 |
| AT4G38130 | Histone deacetylase 19 | defense response to other organism(GO:0098542) | S17 |
| AT4G39950 | cytochrome P450, family 79, subfamily B, polypeptide 2 | defense response by callose deposition in cell wall(GO:0052544) | S17 |
| AT5G01600 | Ferritin-1, chloroplastic | response to external biotic stimulus(GO:0043207) | S17 |
| AT5G02140 | Pathogenesis-related thaumatin superfamily protein | response to external biotic stimulus(GO:0043207) | S17 |
| AT5G02490 | Probable mediator of RNA polymerase II transcription subunit 37c | response to external biotic stimulus(GO:0043207) | S17 |
| AT5G03350 | Lectin-like protein | response to salicylic acid(GO:0009751) | S17 |
| AT5G03780 | TRF-like 10 (Fragment) | response to salicylic acid(GO:0009751) | S17 |
| AT5G04230 | Phenylalanine ammonia-lyase | defense response(GO:0006952) | S17 |
| AT5G04770 | CAT6 | response to external biotic stimulus(GO:0043207) | S17 |
| AT5G05600 | Probable 2-oxoglutarate-dependent dioxygenase At5g05600 | regulation of jasmonic acid mediated signaling pathway(GO:2000022) | S17 |
| AT5G05680 | Nuclear pore complex protein NUP88 | defense response to other organism(GO:0098542) | S17 |
| AT5G06870 | PGIP2 | defense response(GO:0006952) | S17 |
| AT5G07920 | Diacylglycerol kinase 1 | defense response(GO:0006952) | S17 |
| AT5G08370 | Alpha-galactosidase 2 | response to external biotic stimulus(GO:0043207) | S17 |
| AT5G08640 | Flavonol synthase/flavanone 3-hydroxylase | response to external biotic stimulus(GO:0043207) | S17 |
| AT5G10030 | At5g10030 | defense response to bacterium(GO:0042742) | S17 |
| AT5G10380 | E3 ubiquitin-protein ligase RING1 | response to external biotic stimulus(GO:0043207) | S17 |
| AT5G10520 | RBK1 | defense response to other organism(GO:0098542) | S17 |
| AT5G11510 | Transcription factor MYB3R-4 | response to salicylic acid(GO:0009751) | S17 |
| AT5G12170 | Protein CLT3, chloroplastic | defense response to other organism(GO:0098542) | S17 |
| AT5G13330 | Rap2.6L | response to jasmonic acid(GO:0009753) | S17 |
| AT5G13930 | Chalcone synthase family protein | response to jasmonic acid(GO:0009753) | S17 |
| AT5G14740 | Beta carbonic anhydrase 2, chloroplastic | defense response to bacterium(GO:0042742) | S17 |
| AT5G14940 | Protein NRT1/ PTR FAMILY 5.8 | response to external biotic stimulus(GO:0043207) | S17 |
| AT5G15090 | Mitochondrial outer membrane protein porin 3 | defense response to bacterium(GO:0042742) | S17 |
| AT5G16000 | Protein NSP-INTERACTING KINASE 1 | defense response to other organism(GO:0098542) | S17 |
| AT5G18860 | AT5g18860/F17K4_110 | response to jasmonic acid(GO:0009753) | S17 |
| AT5G24090 | Acidic endochitinase | response to external biotic stimulus(GO:0043207) | S17 |
| AT5G24780 | Vegetative storage protein 1 | response to jasmonic acid(GO:0009753) | S17 |
| AT5G25980 | Myrosinase 2 | defense response to insect(GO:0002213) | S17 |
| AT5G27350 | Sugar transporter ERD6-like 17 | response to external biotic stimulus(GO:0043207) | S17 |
| AT5G37260 | Protein REVEILLE 2 | response to salicylic acid(GO:0009751) | S17 |
| AT5G38280 | PR5-like receptor kinase | response to external biotic stimulus(GO:0043207) | S17 |
| AT5G40770 | Prohibitin-3, mitochondrial | salicylic acid biosynthetic process(GO:0009697) | S17 |
| AT5G40910 | Disease resistance protein (TIR-NBS-LRR class) family | defense response(GO:0006952) | S17 |
| AT5G42000 | At5g42000 | defense response to bacterium(GO:0042742) | S17 |
| AT5G42650 | Allene oxide synthase, chloroplastic | jasmonic acid biosynthetic process(GO:0009695) | S17 |
| AT5G44510 | Disease resistance protein TAO1 | defense response to bacterium(GO:0042742) | S17 |
| AT5G46330 | Leucine-rich repeat receptor-like protein kinase (Fragment) | defense response by callose deposition in cell wall(GO:0052544) | S17 |
| AT5G47250 | Probable disease resistance protein At5g47250 | defense response(GO:0006952) | S17 |
| AT5G51600 | 65-kDa microtubule-associated protein 3 | response to external biotic stimulus(GO:0043207) | S17 |
| AT5G54640 | Histone H2A.6 | response to external biotic stimulus(GO:0043207) | S17 |
| AT5G56580 | Mitogen-activated protein kinase kinase 6 | defense response to other organism(GO:0098542) | S17 |
| AT5G58120 | Disease resistance protein (TIR-NBS-LRR class) family | defense response(GO:0006952) | S17 |
| AT5G59780 | Transcription factor MYB59 | response to salicylic acid(GO:0009751) | S17 |
| AT5G60270 | L-type lectin-domain containing receptor kinase I.7 | defense response to bacterium(GO:0042742) | S17 |
| AT5G60890 | Transcription factor MYB34 | defense response to insect(GO:0002213) | S17 |
| AT5G61420 | PMG1 | defense response to bacterium(GO:0042742) | S17 |
| AT5G62470 | Transcription factor MYB96 | response to salicylic acid(GO:0009751) | S17 |
| AT5G63450 | cytochrome P450, family 94, subfamily B, polypeptide 1 | jasmonic acid metabolic process(GO:0009694) | S17 |
| AT5G63770 | Diacylglycerol kinase 2 | defense response(GO:0006952) | S17 |
| AT5G63970 | E3 ubiquitin-protein ligase RGLG3 | defense response to bacterium(GO:0042742) | S17 |
| AT5G64905 | Elicitor peptide 3 | immune system process(GO:0002376) | S17 |
| AT5G65040 | Protein INCREASED RESISTANCE TO MYZUS PERSICAE 1 | defense response to insect(GO:0002213) | S17 |
| AT5G65210 | Transcription factor TGA1 | defense response to bacterium(GO:0042742) | S17 |
| AT5G67160 | Protein ENHANCED PSEUDOMONAS SUSCEPTIBILTY 1 | salicylic acid biosynthetic process(GO:0009697) | S17 |
| AT5G67480 | BTB and TAZ domain protein 4 | response to salicylic acid(GO:0009751) | S17 |
| ATCG00120 | ATP synthase subunit alpha, chloroplastic | defense response to bacterium(GO:0042742) | S17 |
| ATCG00480 | ATP synthase subunit beta, chloroplastic | defense response to fungus(GO:0050832) | S17 |

**Supplementary Table 2: Enrichment of the DEGs for immune functions (along with their significalty represented GO terms as well as GO identifies) in S17 and AtHB8 (vasculature) the SAM cellular populations.**
