# Supplementary material for: Integrated Framework of the Immune-Defense Transcriptional Signatures in the Arabidopsis Shoot Apical Meristem
Source: Int J Mol Sci. 2020 Aug 11;21(16):5745. doi: 10.3390/ijms21165745 (PMC7460820; doi:10.3390/ijms21165745)
Supplement: Supplementary file 1 [file ijms-21-05745-s001.zip › Supplementary material/Supplementary Table 4.docx]

| **GENE ID** | **GENE DESCRIPTION** | **GO TERM** | **CELL POPULATION** |  |
| --- | --- | --- | --- | --- |
| AT1G02450 | NIMIN1 | regulation of immune system process(GO:0002682) | CLV3p |  |
| AT1G07000 | Exocyst subunit Exo70 family protein | regulation of defense response(GO:0031347) | CLV3p |  |
| AT1G17420 | Lipoxygenase 3, chloroplastic | jasmonic acid biosynthetic process(GO:0009695) | CLV3p |  |
| AT1G37130 | Nitrate reductase[NADH] 2 | response to external biotic stimulus(GO:0043207) | CLV3p |  |
| AT1G52400 | Beta-D-glucopyranosyl abscisate beta-glucosidase | defense response to fungus(GO:0050832) | CLV3p |  |
| AT1G61370 | G-type lectin S-receptor-like serine/threonine-protein kinase | innate immune response(GO:0045087) | CLV3p |  |
| AT2G22240 | Inositol-3-phosphate synthase isozyme 2 | defense response to other organism(GO:0098542) | CLV3p |  |
| AT2G27250 | Protein CLAVATA 3 | innate immune response(GO:0045087) | CLV3p |  |
| AT2G35930 | E3 ubiquitin-protein ligase PUB23 | immune effector process(GO:0002252) | CLV3p |  |
| AT2G36910 | ABC transporter B family member 1 | response to external biotic stimulus(GO:0043207) | CLV3p |  |
| AT2G47260 | WRKY transcription factor 23 | response to external biotic stimulus(GO:0043207) | CLV3p |  |
| AT3G44300 | NIT2 | response to external biotic stimulus(GO:0043207) | CLV3p |  |
| AT3G61220 | NAD(P)-binding Rossmann-fold superfamily protein | defense response(GO:0006952) | CLV3p |  |
| AT4G01610 | Cathepsin B-like protease 3 | defense response(GO:0006952) | CLV3p |  |
| AT4G02330 | Probable pectinesterase/pectinesterase inhibitor 41 | response to external biotic stimulus(GO:0043207) | CLV3p |  |
| AT4G24670 | Tryptophan aminotransferase-related protein 2 | defense response to bacterium(GO:0042742) | CLV3p |  |
| AT4G30650 | UPF0057 membrane protein At4g30650 | defense response to fungus(GO:0050832) | CLV3p |  |
| AT5G03210 | Arabidopsis thaliana genomic DNA, chromosome 5, P1 clone:MOK16 | defense response to other organism(GO:0098542) | CLV3p |  |
| AT5G27420 | E3 ubiquitin-protein ligase ATL31 | innate immune response(GO:0045087) | CLV3p |  |
| AT1G31280 | Protein argonaute 2 | defense response to bacterium(GO:0042742) | HDG4 |  |
| AT1G74930 | Ethylene-responsive transcription factor ERF018 | defense response to other organism(GO:0098542) | HDG4 |  |
| AT2G21660 | Glycine-rich RNA-binding protein 7 | innate immune response(GO:0045087) | HDG4 |  |
| AT4G16950 | Disease resistance protein RPP5 | defense response to fungus(GO:0050832) | HDG4 |  |
| AT4G34710 | Arginine decarboxylase 2 | response to jasmonic acid(GO:0009753) | HDG4 |  |
| AT4G39260 | GRP8 | innate immune response(GO:0045087) | HDG4 |  |
| AT5G15380 | DNA (cytosine-5)-methyltransferase DRM1 | defense response to fungus(GO:0050832) | HDG4 |  |
| AT5G65710 | LRR receptor-like serine/threonine-protein kinase HSL2 | defense response to bacterium(GO:0042742) | HDG4 |  |
| AT5G66570 | Oxygen-evolving enhancer protein 1-1, chloroplastic | defense response to bacterium(GO:0042742) | HDG4 |  |
| AT1G11310 | MLO-like protein 2 | defense response to fungus(GO:0050832) | LAS |  |
| AT2G30750 | Cytochrome P450 71A12 | defense response to bacterium(GO:0042742) | LAS |  |
| AT2G46830 | Protein CCA1 | response to jasmonic acid(GO:0009753) | LAS |  |
| AT5G51630 | Disease resistance protein (TIR-NBS-LRR class) family | defense response(GO:0006952) | LAS |  |

| **GENE ID** | **GENE DESCRIPTION** | **GO TERM** | **CELL POPULATION** |
| --- | --- | --- | --- |
| AT1G79090 | Protein PAT1 homolog | innate immune response(GO:0045087) | AtML1 |
| AT2G36890 | Duplicated homeodomain-like superfamily protein | response to jasmonic acid(GO:0009753) | AtML1 |
| AT3G05710 | Syntaxin-43 | regulation of defense response(GO:0031347) | AtML1 |
| AT4G14720 | TIFY domain/Divergent CCT motif family protein | regulation of defense response(GO:0031347) | AtML1 |
| AT5G08280 | Porphobilinogen deaminase, chloroplastic | response to external biotic stimulus(GO:0043207) | AtML1 |
| AT1G02360 | Chitinase family protein | response to external biotic stimulus(GO:0043207) | HMG |
| AT1G13230 | Piriformospora indica-insensitive protein 2 | response to external biotic stimulus(GO:0043207) | HMG |
| AT1G55020 | Linoleate 9S-lipoxygenase 1 | response to jasmonic acid(GO:0009753) | HMG |
| AT1G70130 | Putative L-type lectin-domain containing receptor kinase V.2 | defense response to other organism(GO:0098542) | HMG |
| AT1G71400 | Receptor-like protein 12 | defense response(GO:0006952) | HMG |
| AT1G72140 | Protein NRT1/ PTR FAMILY 5.12 | response to external biotic stimulus(GO:0043207) | HMG |
| AT1G79380 | E3 ubiquitin-protein ligase RGLG4 | response to jasmonic acid(GO:0009753) | HMG |
| AT2G02220 | Phytosulfokine receptor 1 | regulation of defense response(GO:0031347) | HMG |
| AT2G24570 | WRKY transcription factor 17 | defense response to bacterium(GO:0042742) | HMG |
| AT2G26380 | Leucine-rich repeat (LRR) family protein | defense response(GO:0006952) | HMG |
| AT2G28790 | Pathogenesis-related thaumatin superfamily protein | response to external biotic stimulus(GO:0043207) | HMG |
| AT2G28900 | Outer envelope pore protein 16-1, chloroplastic | response to jasmonic acid(GO:0009753) | HMG |
| AT2G33050 | Receptor like protein 26 | defense response(GO:0006952) | HMG |
| AT3G14840 | Probable leucine-rich repeat receptor-like serine/threonine-protein kinase | regulation of innate immune response(GO:0002682) | HMG |
| AT3G18690 | Protein MKS1 | regulation of innate immune response(GO:0002682) | HMG |
| AT4G31550 | Probable WRKY transcription factor 11 | defense response to bacterium(GO:0042742) | HMG |
| AT4G32650 | KC1 | response to external biotic stimulus(GO:0043207) | HMG |
| AT5G05190 | Protein ENHANCED DISEASE RESISTANCE 4 | regulation of response to biotic stimulus(GO:0002831) | HMG |
| AT5G10720 | Histidine kinase 5 | response to external biotic stimulus(GO:0043207) | HMG |
| AT5G23820 | MD-2-related lipid-recognition protein 3 | defense response(GO:0006952) | HMG |
| AT5G44070 | PCS1 | defense response to bacterium(GO:0042742) | HMG |
| AT5G45510 | Probable disease resistance protein At5g45510 | defense response(GO:0006952) | HMG |
| AT5G47910 | Respiratory burst oxidase homolog protein D | defense response to fungus(GO:0050832) | HMG |
| AT5G57220 | Cytochrome P450 81F2 | defense response to fungus(GO:0050832) | HMG |
| AT5G61890 | Ethylene-responsive transcription factor ERF114 | defense response to fungus(GO:0050832) | HMG |
| AT5G61910 | DCD (Development and Cell Death) domain protein | regulation of defense response(GO:0031347) | HMG |

| **GENE ID** | **GENE DESCRIPTION** | GO TERM | **CELL**  **POPULATION** |
| --- | --- | --- | --- |
| AT1G02450 | NIMIN1 | regulation of immune system process(GO:0002682) | FILp |
| AT1G09750 | Aspartyl protease AED3 | defense response to other organism(GO:0098542) | FILp |
| AT1G20510 | 4-coumarate--CoA ligase-like 5 | jasmonic acid biosynthetic process(GO:0009695) | FILp |
| AT1G28480 | Glutaredoxin-C9 | response to jasmonic acid(GO:0009753) | FILp |
| AT1G33970 | Immune-associated nucleotide-binding protein 9 | regulation of innate immune response(GO:0002682) | FILp |
| AT1G72840 | Disease resistance protein (TIR-NBS-LRR class) | defense response(GO:0006952) | FILp |
| AT1G73620 | Pathogenesis-related thaumatin superfamily protein | response to external biotic stimulus(GO:0043207) | FILp |
| AT1G73805 | Protein SAR DEFICIENT 1 | regulation of immune system process(GO:0002682) | FILp |
| AT1G80840 | Probable WRKY transcription factor 40 | regulation of defense response to virus by host(GO:0050691) | FILp |
| AT2G17660 | At2g17660 | regulation of immune system process(GO:0002682) | FILp |
| AT2G26440 | Probable pectinesterase/pectinesterase inhibitor 12 | response to external biotic stimulus(GO:0043207) | FILp |
| AT2G27080 | NDR1/HIN1-like protein 13 | defense response to bacterium(GO:0042742) | FILp |
| AT2G41370 | Regulatory protein NPR5 | induced systemic resistance, jasmonic acid mediated signaling pathway(GO:0009864) | FILp |
| AT3G10930 | Uncharacterized protein At3g10930 | response to external biotic stimulus(GO:0043207) | FILp |
| AT3G25250 | Serine/threonine-protein kinase OXI1 | defense response(GO:0006952) | FILp |
| AT3G57130 | Regulatory protein NPR6 | induced systemic resistance, jasmonic acid mediated signaling pathway(GO:0009864) | FILp |
| AT4G39830 | At4g39830 | regulation of defense response(GO:0031347) | FILp |
| AT5G14930 | Senescence-associated carboxylesterase 101 | regulation of defense response(GO:0031347) | FILp |
| AT5G21150 | Protein argonaute 9 | immune effector process(GO:0002252) | FILp |
| AT5G48485 | Putative lipid-transfer protein DIR1 | innate immune response(GO:0045087) | FILp |
| AT5G48490 | At5g48490 | defense response to other organism(GO:0098542) | FILp |
| AT5G49520 | Probable WRKY transcription factor 48 | defense response to bacterium(GO:0042742) | FILp |
| AT5G64570 | Beta-D-xylosidase 4 | innate immune response(GO:0045087) | FILp |
| AT1G02205 | Fatty acid hydroxylase superfamily | defense response to fungus(GO:0050832) | KAN1 |
| AT1G71030 | At1g71030/F23N20_2 | response to jasmonic acid(GO:0009753) | KAN1 |
| AT1G73620 | Pathogenesis-related thaumatin superfamily protein | response to external biotic stimulus(GO:0043207) | KAN1 |
| AT1G75830 | Defensin-like protein 13 | defense response(GO:0006952) | KAN1 |
| AT2G46410 | Transcription factor CPC | response to jasmonic acid(GO:0009753) | KAN1 |
| AT3G01500 | Beta carbonic anhydrase 1, chloroplastic | defense response to bacterium(GO:0042742) | KAN1 |
| AT3G13790 | Beta-fructofuranosidase, insoluble isoenzyme CWINV1 | defense response to fungus(GO:0050832) | KAN1 |
| AT3G20820 | Leucine-rich repeat (LRR) family protein | defense response(GO:0006952) | KAN1 |
| AT3G25180 | Cytochrome P450 82G1 | defense response(GO:0006952) | KAN1 |
| AT3G26470 | Powdery mildew resistance protein, RPW8 domain-containing protein | defense response to fungus(GO:0050832) | KAN1 |
| AT3G45140 | Lipoxygenase 2, chloroplastic | response to jasmonic acid(GO:0009753) | KAN1 |
| AT4G02410 | L-type lectin-domain containing receptor kinase IV.3 | defense response to bacterium(GO:0042742) | KAN1 |
| AT4G23670 | AT4G23670 protein | defense response to bacterium(GO:0042742) | KAN1 |
| AT4G30650 | UPF0057 membrane protein At4g30650 | defense response to fungus(GO:0050832) | KAN1 |
| AT5G14740 | Beta carbonic anhydrase 2, chloroplastic | defense response to bacterium(GO:0042742) | KAN1 |
| AT1G65390 | Protein PHLOEM PROTEIN 2-LIKE A5 | defense response(GO:0006952) | WUS |
| AT1G73805 | Protein SAR DEFICIENT 1 | positive regulation of response to biotic stimulus(GO:0002833) | WUS |
| AT3G29160 | Non-specific serine/threonine protein kinase | regulation of immune effector process(GO:0002697) | WUS |
| AT5G39580 | Peroxidase 62 | defense response to fungus(GO:0050832) | WUS |
| AT5G52450 | Protein DETOXIFICATION | response to external biotic stimulus(GO:0043207) | WUS |
