# Supplementary material for: Integrated Framework of the Immune-Defense Transcriptional Signatures in the Arabidopsis Shoot Apical Meristem
Source: Int J Mol Sci. 2020 Aug 11;21(16):5745. doi: 10.3390/ijms21165745 (PMC7460820; doi:10.3390/ijms21165745)
Supplement: Supplementary file 1 [file ijms-21-05745-s001.zip › Supplementary material/Supplementary Table 3_overlap between genes of CLV3p and flg22.docx]

**Supplementary Table 3: Overlap among the DEGs of CLV3p and flg22**

**7 common elements in "flg22 URGs" and "ClV3p DRGs":**

AT1G08930: a putative sucrose transporter whose gene expression is induced by dehydration and cold

AT2G28305: Cytokinin riboside 5'-monophosphate phosphoribohydrolase LOG1

AT1G58340: Functions as a multidrug and toxin extrusion transporter.

AT5G66320: Encodes GATA transcription factor gene GNC, regulating carbon and nitrogen metabolism

AT1G14880: Protein PLANT CADMIUM RESISTANCE 1;

AT3G12700: Involved in the maintenance of the shoot apical meristem

AT4G14365: Putative E3 ubiquitin-protein ligase XBAT34

**4 common elements in "flg22 URGs" and "CLV3p-URGs":**

AT4G37370: Cytochrome P450, family 81, subfamily D, polypeptide 8;

AT3G25610: haloacid dehalogenase-like hydrolase family protein; Involved in transport of phospholipids

AT2G32190: Cysteine-rich/transmembrane domain A-like protein

AT2G32190: Cysteine-rich/transmembrane domain A-like protein
