# Supplementary figures and images for: Integrated Framework of the Immune-Defense Transcriptional Signatures in the Arabidopsis Shoot Apical Meristem
Source: Int J Mol Sci. 2020 Aug 11;21(16):5745. doi: 10.3390/ijms21165745 (PMC7460820; doi:10.3390/ijms21165745)

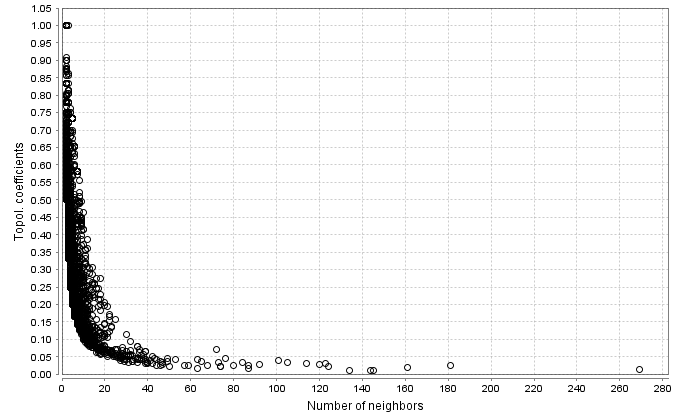

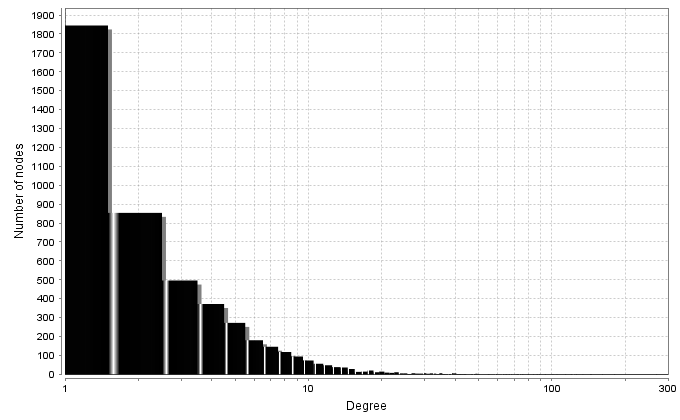

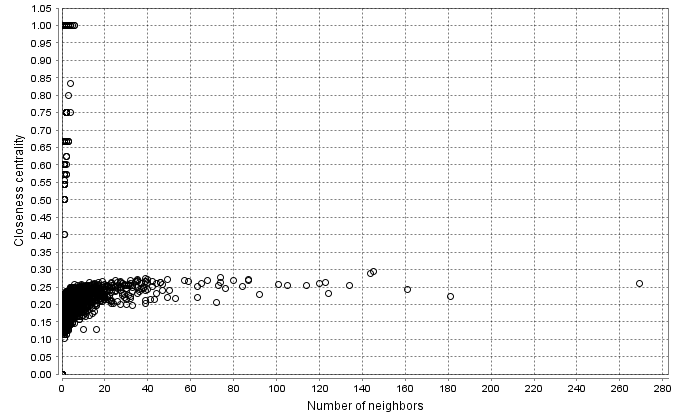

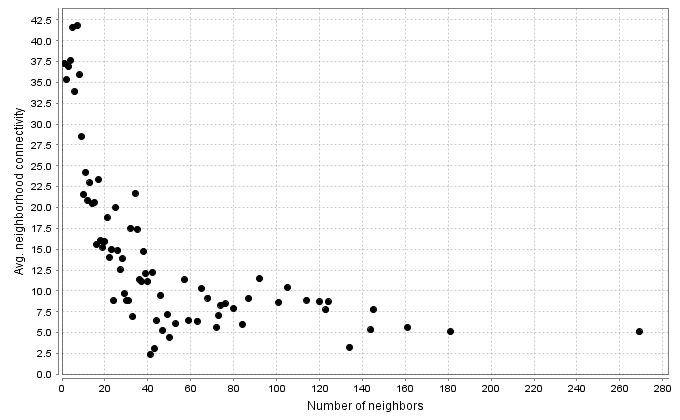

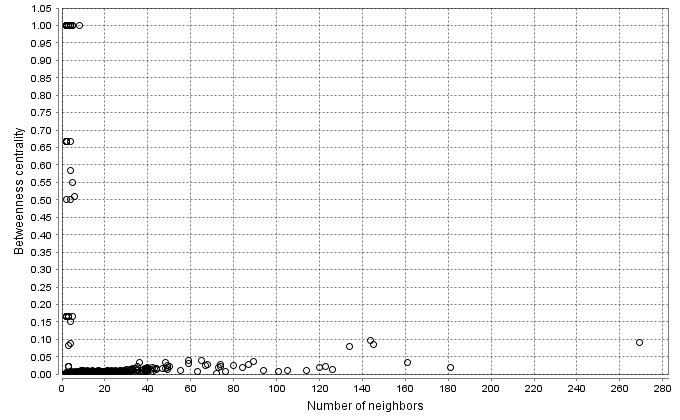

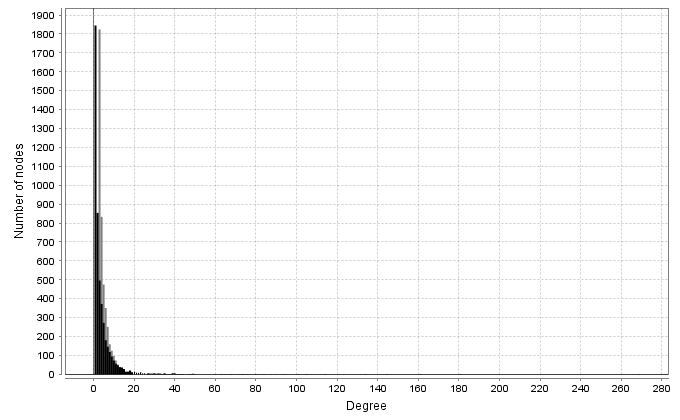

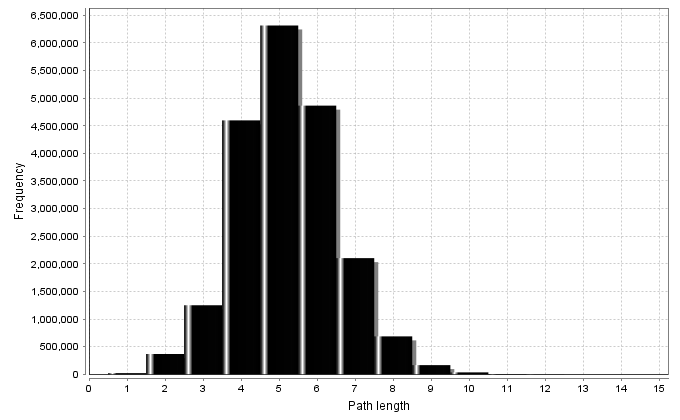


**Supplementary Figure 1: Topological analysis of the SAM-interactome network**

Supplement: Supplementary file 1 [file ijms-21-05745-s001.zip › Supplementary material/Supplementary Figure 1.docx]
